# The impact of probiotics on oxidative stress and inflammatory markers in patients with diabetes: a meta-research of meta-analysis studies

**DOI:** 10.3389/fnut.2025.1552358

**Published:** 2025-03-07

**Authors:** Xi Chen, Lijun Yan, Jie Yang, Chenlong Xu, Lv Yang

**Affiliations:** ^1^Department of Endocrinology, The Affiliated Taizhou People's Hospital of Nanjing Medical University, Taizhou School of Clinical Medicine, Nanjing Medical University, Taizhou, China; ^2^Department of Geriatric Gastroenterology, The First Affiliated Hospital with Nanjing Medical University, Nanjing Medical University, Nanjing, China; ^3^Department of Laboratory Medicine, Ningbo Yinzhou No.2 Hospital, Ningbo, China

**Keywords:** probiotics, oxidative stress, inflammation, metabolic disorders, biomarkers

## Abstract

**Objective:**

Probiotic supplementation has gained attention for its potential to modulate inflammatory and oxidative stress biomarkers, particularly in metabolic disorders. This meta-analysis evaluates the effects of probiotics on C-reactive protein (CRP), tumor necrosis factor-alpha (TNF-*α*), interleukin-6 (IL-6), malondialdehyde (MDA), total antioxidant capacity (TAC), glutathione (GSH), and nitric oxide (NO) in patients with diabetes.

**Methods:**

A Meta-Research was conducted on 15 meta-analyses of unique 33 randomized controlled trials (RCTs) published between 2015 and 2022, involving 26 to 136 participants aged 26 to 66 years. Data were synthesized using standardized mean differences (SMD), with sensitivity analysis using a random-effect model.

**Results:**

Probiotic supplementation significantly reduced CRP (SMD = −0.79, 95% CI: −1.19, −0.38), TNF-*α* (SMD = −1.35, 95% CI: −2.05, −0.66), and MDA levels (WMD: -0.82, 95% CI: −1.16, −0.47). Probiotics increased GSH (SMD = 1.00, 95% CI: 0.41, 1.59), TAC (SMD = 0.48, 95% CI: 0.27, 0.69), and NO (SMD = 0.60, 95% CI: 0.30, 0.91). Result on IL-6 was not significant (SMD = −0.29, 95% CI: −0.66, 0.09). Sensitivity analyses confirmed robustness.

**Conclusion:**

Probiotics significantly improved inflammatory and oxidative stress biomarkers in patients with diabetes, with variations influenced by population and dosage. Future studies should explore novel probiotic strains and longer interventions.

## 1 Introduction

Diabetes mellitus (DM) is a chronic metabolic disorder that has reached epidemic proportions worldwide, affecting approximately 537 million adults as of 2021 (1). This rate is increasing and imposing a significant burden on healthcare systems globally. One of the major challenges in diabetes management is addressing the complications arising from chronic low-grade inflammation and oxidative stress, both of which play critical roles in the progression of diabetes-related complications such as nephropathy and cardiovascular diseases (2). Oxidative stress, characterized by an imbalance between reactive oxygen species (ROS) production and antioxidant defenses, is a hallmark of diabetes (3). Inflammation and oxidative stress are linked, with elevations in one often exacerbating the other in a mutually reinforcing cycle (4). Addressing these interconnected pathways has become a crucial aspect of diabetes care.

Probiotics, defined as live microorganisms that provide health benefits when consumed in adequate amounts, have emerged as a promising intervention in diabetes management (1, 5). The primary mechanisms of action of probiotics involve improving gut microbiota composition, strengthening intestinal barrier integrity, and reducing systemic endotoxemia by lowering lipopolysaccharide (LPS) translocation (6). These mechanisms ultimately lead to decreased activation of inflammatory pathways and enhanced antioxidant defenses. Probiotics also stimulate the production of short-chain fatty acids (SCFAs), which can modulate immune responses and improve glucose metabolism (7, 8).

Despite the growing interest in probiotics, existing research on their effects on oxidative stress and inflammatory markers in patients with diabetes has yielded inconsistent results. Regarding inflammation, while some meta-analysis studies have reported no significant effect of probiotics on C-reactive protein (CRP) levels in patients with diabetes (9, 10), others have demonstrated a significant reduction (11, 12). This could be attributed to differences in statistical analyses and the heterogeneity of studies included in the referenced meta-analyses. This also applies to antioxidant markers such as glutathione (GSH), where the study results remain inconsistent (13, 14). In the current umbrella review, we aimed to provide a comprehensive evaluation of all existing meta-analyses on the effects of probiotics in diabetic patients. To ensure robustness, we systematically assessed all studies included in these meta-analyses to determine whether they met our predefined inclusion and exclusion criteria. Studies that fulfilled these criteria were incorporated into our analysis. Additionally, we identified studies from our systematic search that were not part of the included meta-analyses but met the eligibility criteria and incorporated them as well. This meticulous approach minimized the likelihood of missing relevant studies. By comparing our findings with previous research, performing comprehensive statistical analyses, and evaluating the evidence using the GRADE framework, we sought to provide a definitive conclusion on the efficacy of probiotics in modulating inflammation among diabetic patients. This integrated approach enhances the reliability of our results and contributes to a deeper understanding of the potential role of probiotics in diabetes management.

This meta-analysis aims to evaluate the effects of probiotic supplementation on key biomarkers of oxidative stress (malondialdehyde (MDA), total antioxidant capacity (TAC), GSH, and nitric oxide [NO]) and inflammation (interleukin-6 (IL-6), tumor necrosis factor-alpha (TNF-*α*), and CRP) in individuals with diabetes. By synthesizing evidence from clinical trial studies conducted exclusively on patients with diabetes, this study seeks to provide a robust assessment of the therapeutic potential of probiotics in mitigating oxidative and inflammatory complications in this high-risk population.

## 2 Methods

### 2.1 Protocol and guidelines

This umbrella review and meta-analysis aimed to evaluate the effects of probiotics on inflammatory markers and oxidative stress in diabetic patients by assessing existing meta-analyses and systematic reviews, as well as conducting a comprehensive meta-analysis of randomized controlled trials (RCTs). This meta-analysis was conducted in accordance with the Preferred Reporting Items for Systematic Reviews and Meta-Analyses (PRISMA) guidelines (15). The study protocol has been registered in the International Prospective Register of Systematic Reviews (PROSPERO) under the registration number CRD42023229865.

### 2.2 Search strategy

To identify relevant studies, a comprehensive search was conducted in the following databases: PubMed, Scopus, Web of Science, EMBASE, and Cochrane Central Library, up to November 2024. The search terms included a combination of Medical Subject Headings (MeSH) and keywords: (“Probiotics” OR “Saccharomyces” OR “Lactobacillus” OR “Bifidobacterium”) AND (“Oxidative Stress” OR “Total Antioxidant Capacity” OR “TAC” OR “Antioxidant” OR “Oxidant” OR “Reactive oxygen species” OR “Malondialdehyde” OR “MDA OR “Glutathione” OR “GSH” OR “Nitric Oxide”).

To increase sensitivity, wildcard terms (e.g., “*”) were used. The search was restricted to English-language publications. Additionally, the reference lists of the included meta-analysis studies were reviewed to identify any RCTs that met the predefined inclusion and exclusion criteria. Studies meeting these criteria were incorporated into the analysis to ensure a comprehensive assessment and minimize the risk of missing relevant trials. In addition to peer-reviewed articles, we conducted a comprehensive search for gray literature and unpublished studies by exploring relevant conference proceedings, theses, dissertations, and clinical trial registries to minimize publication bias and ensure a thorough inclusion of all pertinent data.

### 2.3 Inclusion and exclusion criteria

The following PICOS criteria were applied for study selection: Population (P): Adults aged ≥18 years with diabetes mellitus; Intervention (I): Probiotic supplementation; Comparison (C): Placebo or control group; Outcomes (O): Oxidative stress biomarkers including MDA, TAC, GSH, and NO, and inflammatory markers such as IL-6, TNF-*α*, and CRP; Study design (S): Systematic review, meta-analysis, as well as RCT studies, providing effect sizes and corresponding confidence intervals (CI) for each outcome. Exclusion criteria included *in vitro* or *in vivo* studies, observational studies, case reports, quasi-experimental studies, and meta-analysis studies lacking sufficient data for effect size calculation.

### 2.4 Quality assessment

The methodological quality of the included meta-analysis studies was assessed independently by two reviewers using the Measurement Tool to Assess Systematic Reviews (*AMSTAR*) *2* checklist (16). *AMSTAR2* evaluates both critical and non-critical domains, such as protocol registration, risk of bias assessment, and adherence to statistical best practices in meta-analyses. Reviews with high-quality methodology were considered more reliable, while those with significant flaws were excluded. Discrepancies were resolved through discussion or consultation with a senior author. Studies scoring ≥7 were categorized as high-quality.

The methodological quality of the included RCTs was evaluated using the Cochrane Risk of Bias Tool. This tool assesses seven domains including random sequence generation, allocation concealment, blinding of participants and personnel, blinding of outcome assessment, incomplete outcome data, selective reporting, and other sources of bias. Each domain was classified as low, high, or unclear risk, with an overall risk of bias assigned to each study to ensure a rigorous quality assessment (17). Discrepancies were resolved through discussion or consultation with a senior author.

### 2.5 Data extraction

Two independent reviewers screened titles and abstracts, followed by full-text evaluation of eligible studies. Extracted data from meta-analysis and RCT studies included: study characteristics (author, publication year, location), participant details (sample size, age, health status), intervention specifics (probiotic type, dosage, and duration), and outcomes (effect sizes (ESs) with 95% confidence intervals [CIs] for MDA, GSH, TAC, NO, IL-6, TNF-*α*, and CRP). Disagreements were resolved through discussion with a third reviewer.

### 2.6 Statistical analysis

Data were synthesized using effect sizes (SMD) and corresponding 95% confidence intervals. Random-effects models were applied for pooling data. Heterogeneity was assessed using the Cochran’s Q test and *I^2^* statistic (*I^2^* > 50%, *p* < 0.1). Subgroup analyses were performed to explore heterogeneity based on variables such as sample size, probiotic type, intervention duration, and population characteristics. Sensitivity analyses assessed the robustness of the findings by excluding individual studies. To address heterogeneity beyond subgroup and sensitivity analyses, we conducted meta-regression analyses to explore potential sources of variability among studies. Specifically, we examined the effects of moderator variables such as intervention duration and sample size on the observed outcomes. Publication bias was evaluated using funnel plots (for markers with >10 included studies) and statistical tests [Begg’s (18) and Egger’s tests (19)]. If bias was detected, a trim-and-fill method was applied. All statistical analyses were conducted using STATA software version 16.0 (StataCorp, College Station, TX, United States), with a significance threshold of *p* < 0.05.

### 2.7 GRADE assessment

The quality of the evidence was evaluated using the GRADE (Grading of Recommendations, Assessment, Development, and Evaluations) framework (20). This framework takes into account several factors to determine the overall confidence in the effect estimates, including risk of bias, inconsistency, indirectness, imprecision, and publication bias. The risk of bias in each included study was assessed using the Cochrane Risk of Bias tool. Studies with a high risk of bias were downgraded in terms of evidence quality. Heterogeneity across the studies was assessed using the I^2^ statistic. Significant heterogeneity (I^2^ > 50%) indicated inconsistency in study results, which could lead to a downgrading of the evidence. Indirectness was considered by evaluating whether the study populations, interventions, and outcomes were directly applicable to the research question. Studies with populations or interventions that did not align closely with the review’s focus were downgraded for indirectness. The precision of the effect estimates was assessed by the width of the confidence intervals (CIs). Wide CIs, suggesting greater uncertainty in the estimates, led to a downgrade in the quality of evidence. To evaluate publication bias, funnel plots and statistical tests were used. If publication bias was detected, the evidence quality was downgraded.

After these assessments, the quality of evidence for each outcome was categorized as high, moderate, low, or very low, helping to clarify the strength of the evidence supporting the effects of probiotics on inflammatory and oxidative stress biomarkers in diabetic patients.

## 3 Results

### 3.1 Systematic review of meta-analysis studies

The flow diagram of the study selection process for meta-analysis studies is illustrated in Supplementary Figure S1. A systematic search of electronic databases resulted in a total number of 709 articles. After removing the duplicate articles (*n* = 94), 615 articles were screened by reading their titles and abstracts, leading to 18 articles whose full texts were evaluated. Ultimately, 15 meta-analyses were included in the systematic review (9–14, 21–29).

Study population were patients with type 2 and 1-diabetes mellitus (T2DM and T1DM), diabetic nephropathy, gestational diabetes mellitus (GDM), and prediabetes. *Lactobacillus*, *Bifidobacterium*, and *Streptococcus* being the most commonly used strains. The quality of the included studies, assessed using tools such as the Cochrane Risk of Bias Tool and the Jadad scale, revealed that most studies were of high quality (Supplementary Table S1). The quality of the included studies was assessed based on a series of quality criteria (Q1–Q16) defined by the *AMSTAR 2* checklist and categorized as high or moderate (Supplementary Table S2). Most studies in this review were assessed as having moderate quality (12 of 15 included studies). Among these, the studies by Ardeshirlarijan et al. (24), Zheng et al. (26), Naseri et al. (23) were classified as high quality, meeting most of the criteria with minimal biases. Notably, the study by Naseri et al. (23) exhibited the highest quality, fulfilling all assessment criteria.

The systematic review of meta-analyses revealed that probiotics, particularly *Lactobacillus*, *Bifidobacterium*, and *Streptococcus*, significantly reduced inflammatory markers like CRP (9 of 11 studies) and TNF-*α* (3 of 4 studies), as well as oxidative stress markers such as MDA (9 of 10 studies), while improving antioxidant levels including GSH (7 of 10 studies) and TAC (7 of 9 studies) in diabetic populations. However, the effects on NO and IL-6 were inconsistent, with several studies reporting no significant changes (7 of 11 and 3 of 5 studies, respectively) (Supplementary Table S1).

### 3.2 Study selection process of RCTs

**Figure 1:**
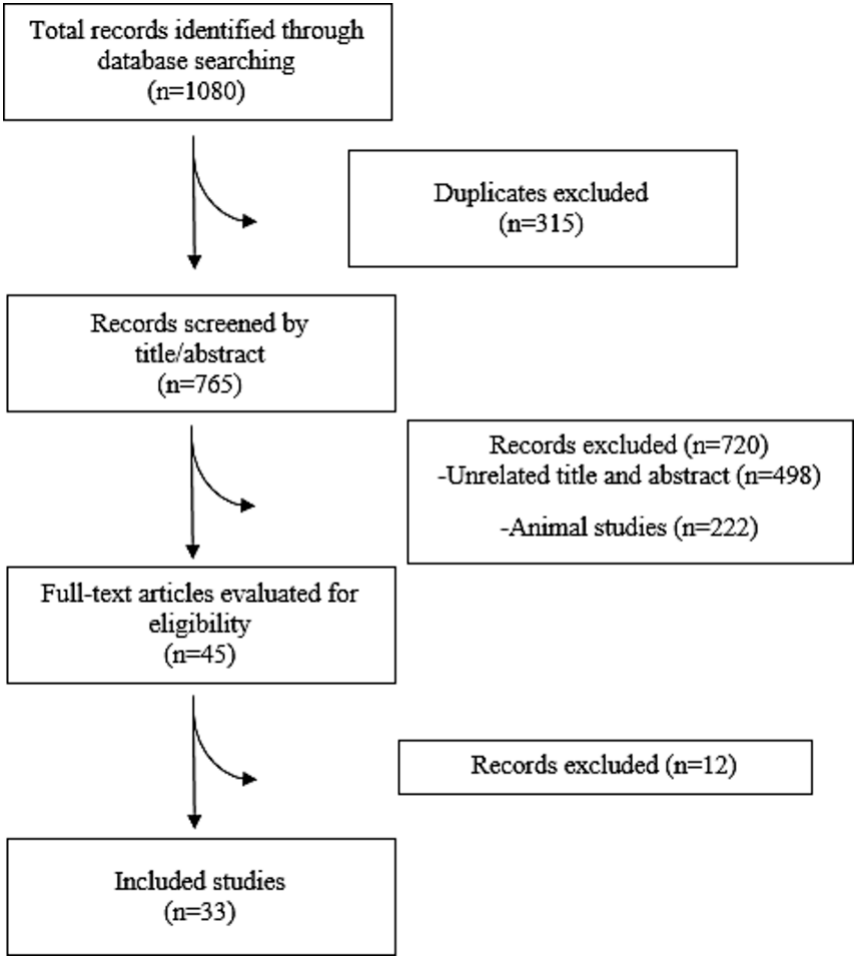
Flow diagram of study selection.

Initially, 978 records were identified through systematic database searches. Additionally, 102 unique RCTs cited within 15 meta-analysis studies were added after the initial search to ensure comprehensive inclusion. Following the removal of duplicate articles and exclusion of studies that did not meet the inclusion criteria, a total of 33 RCTs were included in the analysis (30–62) (Figure 1). The characteristics of the included RCTs are summarized in Table 1.

**Table 1:** Study characteristics of included RCTs.

Study, country	Design	Sample Size	Intervention	Control	Sex	Health conditions	Age intervention (years)	Age control (years)	Duration (weeks)
Mazruei Arani et al. (49), Iran	Parallel, R, DB	60	Honey containing heat-resistant probiotic *Bacillus coagulans* T4 (10^8^ CFU/g)	Control honey	F/M	DN	62.7 ± 9.1	60.3 ± 8.5	12
Mafi et al. (47), Iran	Parallel, R, PC, DB	60	*Lactobacillus acidophilus* ZT-L1, *Bifidobacterium bifidum* ZT-B1, *Lactobacillus reuteri* ZT-Lre, and *Lactobacillus fermentum* ZT-L3 (8 × 10^9^ CFU/d)	Placebo	F/M	DN	58.9 ± 8.8	60.9 ± 4.4	12
Soleimani et al. (58), Iran	Parallel, R, PC, DB	60	*Lactobacillus acidophilus*, *Lactobacillus casei*, *Bifidobacterium bifidum* (6 × 10^9^ CFU /d)	Placebo	F/M	Diabetic hemodialysis	54 ± 16	59.4 ± 16	12
Mohseni et al. (53), Iran	Parallel, R, PC, DB	60	*Lactobacillus acidophilus, Lactobacillus casei, Lactobacillus Fermentum* and *Bifidobacterium bifidum* (8 × 10 *9)”	Placebo	F/M	Diabetic foot ulcer	62.6 ± 9.7	58.5 ± 11	12
Raygan et al. (54), Iran	Parallel, R, PC, DB	60	*Bifidobacterium bifidum, Lactobacillus casei, Lactobacillus acidophilus* (6 × 10^9^ CFU/d)	Placebo	F/M	T2DM with coronary heart disease	60.7 ± 9.4	61.8 ± 9.8	12
Miraghajani et al. (50), Iran	Parallel, R, SB	40	Soy milk containing *Lactobacillus plantarum* A7 (2 × 10^7^ CFU/mL)	Control soy milk	F/M	DN	56.9 ± 1.81	53.6 ± 1.6	8
Tonucci et al. (62), Brazil	Parallel, R, PC, DB	45	Fermented milk containing *Lactobacillus acidophilus* La-5 and *Bifidobacterium animalis* subsp. lactis BB-12 (2 × 10^9^ CFU/d)	Conventional fermented milk	F/M	T2DM	51.83 ± 6.64	50.95 ± 7.2	6
Asemi et al., 2015 (31), Iran	Cross-over, R, DB	102	Beta-carotene fortified synbiotic containing *Lactobacillus sporogenes* (1 × 10^7^ CFU), 0.1 g inulin and 0.05 g beta-carotene	Same food without probiotic, inulin, and beta-carotene	F/M	T2DM	52.9 ± 8.1	52.9 ± 8.1	6
Bahmani et al. (36) (A), Iran	Cross-over, R, DB	54	Bread containing *Lactobacillus sporogenes* (1 × 10^8^ CFU)	Control bread	F/M	T2DM	51.3 ± 10.4	53.4 ± 7.5	8
Bahmani et al. (36) (B), Iran	Cross-over, R, DB	54	Synbiotic bread: *Lactobacillus sporogenes* (1 × 10^8^ CFU) and 0.07 g inulin/g	Control bread	F/M	T2DM	52.0 ± 7.2	53.4 ± 7.6	8
Asemi et al. (32), Iran	Cross-over, R, DB	124	The synbiotic food containing 27 × 10^7^ CFU *Lactobacillus sporogenes* and 1.08 g inulin	Control food	F/M	Diabetes	51.3 ± 8.7	51.3 ± 8.7	6
Asemi et al. (33), Iran	Parallel, R, DB	54	*Lactobacillus acidophilus* (2 × 10^9^ CFU), *Lactobacillus casei* (7 × 10^9^ CFU), *Lactobacillus rhamnosus* (1.5 × 10^9^ CFU), *Lactobacillus bulgaricus* (2 × 10^8^ CFU), *Bifidobacterium breve* (2 × 10^10^ CFU), *Bifidobacterium longum* (7 × 10^9^ CFU), *Streptococcus thermophilus* (1.5 × 10^9^ CFU), and 100 mg fructo-oligosaccharide	Placebo	F/M	T2DM	50.51 ± 9.82	52.59 ± 7.14	8
Ejtahed et al. (38), Iran	Parallel, R, DB	60	Probiotic yogurt containing *Lactobacillus acidophilus* La5 and *Bifidobacterium lactis* Bb12 (7.23 × 10 6 and 6.04 × 10 6 CFU/g, respectively)	Conventional yogurt	F/M	T2DM	50.87 ± 7.68	51.00 ± 7.32	6
Mazloom et al. (48), Iran	Parallel, R, SB	34	*Lactobacillus acidophilus*, *Lactobacillus bulgaricus*, *Lactobacillus bifidum, and Lactobacillus casei*	Placebo	F/M	T2DM	55.4 ± 8	51.8 ± 10.2	6
Hajifaraji et al. (41), Iran	Parallel, R, DB	56	*Lactobacillus acidophilus* LA-5, *Bifidobacterium* BB-12, *Streptococcus Thermophilus* STY-31 *and Lactobacillus delbrueckii bulgaricus* LBY-27 (>4 × 10^9^ CFU)	Placebo	F	GDM	28.14 ± 6.24	26.48 ± 5.23	8
Jamilian et al. (45), Iran	Parallel, R, DB	57	*Lactobacillus acidophilus*, *Bifidobacterium bifidum*, *Lactobacillus reuteri*, and *Lactobacillus fermentum* (8 × 10^9^ CFU/g)	Placebo	F	GDM	31.2 ± 5.9	29.9 ± 3.7	6
Badehnoosh et al. (35), Iran	Parallel, R, DB	60	*Lactobacillus acidophilus*, *Lactobacillus casei*, and *Bifidobacterium bifidum* (6 × 109 CFU/g)	Placebo	F	GDM	28.8 ± 5.4	27.8 ± 3.7	6
Jafarnejad et al. (44), Iran	Parallel, R, DB	72	*Streptococcus thermophilus*, *Bifidobacterium breve*, *Bifidobacterium longum*, *Bifidobacterium infantis*, *Lactobacillus acidophilus*, *Lactobacillus plantarum*, *Lactobacillus paracasei*, and *Lactobacillus delbrueckii* subsp. Bulgaricus (112.5 × 10^9^ CFU)	Placebo	F	GDM	32.4 ± 3.1	31.9 ± 4.0	8
Babadi et al. (34), Iran	Parallel, R, DB	48	*Lactobacillus acidophilus, Lactobacillus casei, Bifidobacterium bifidum, Lactobacillus fermentum* (8 × 10^9^ CFU/g)	Placebo	F	GDM	28.3 ± 4.3	29.0 ± 4.2	6
Andreasen et al. (30), Denmark	Parallel, R, DB	45	*Lactobacillus acidophilus* NCFM (10^10^ CFU/g)	Placebo	F/M	T2DM	55	60	4
Bayat et al. (37), Iran	Parallel, RCT	40	Probiotic yogurt (150 g/d)	Dietary advice	F/M	T2DM	54.1 ± 9.54	46.95 ± 9.34	8
Feizollahzadeh et al. (39), Iran	Parallel, R, DB	40	Soy milk containing *Lactobacillus planetarum* A7 (2 × 10^7^ CFU)	Pure soy milk	F/M	T2DM	56.90 ± 1.81	53.6 ± 1.6	8
Firouzi et al. (40), Malaysia	Parallel, R, DB	136	*Lactobacillus acidophilus, Lactobacillus* *casei, Lactobacillus lactis, Bifidobacterium bifidum, Bifidobacterium longum* and *Bifidobacterium infantis* (3 × 10^10^ CFU)	Placebo	F/M	T2DM	52.9 ± 9.2	54.2 ± 8.3	12
Mobini et al. (51) (A), Sweden	Parallel, R, DB	30	*Lactobacillus reuteri* DSM 17938 (10^8^ CFU/d)	Placebo	F/M	T2DM	66 ± 6	65 ± 5	12
Mobini et al. (51) (B), Sweden	Parallel, R, DB	29	*Lactobacillus reuteri* DSM 17938 (10^10^ CFU/d)	Placebo	F/M	T2DM	64 ± 6	66 ± 5	12
Mohamadshahi et al. (52), Iran	Parallel, R, DB	44	probiotic yogurt containing of both *Lactobacillus acidophilus* La-5 and *Bifidobacterium lactis* Bb-12 (7.4 × 10^6^ CFU/mg)	Conventional yogurt	F/M	T2DM	53.00 ± 5.9	49.00 ± 7.08	8
Sabico et al. (56), UK	Parallel, R, DB	61	*Bifidobacterium bifidum* W23, *Bifidobacterium lactis* W52, *Lactobacillus acidophilus* W37, *Lactobacillus brevis* W63, *Lactobacillus casei* W56, *Lactobacillus salivarius* W24, *Lactococcus* *lactis* W19 and *Lactococcus lactis* W58 (2.5 × 10^9^ CFU/g)	Placebo	F/M	T2DM	48.0 ± 8.3	46.6 ± 5.9	24
Sato et al. (57), Japan	Parallel, R	68	*Lactobacillus casei strain* Shirota-fermented milk (4 × 10^10^ CFU)	No probiotics	F/M	T2DM	64.0 ± 9.2	65.0 ± 8.3	16
Tajadadi-Ebrahimi et al. (59), Iran	Parallel, R, DB	54	Probiotic bread contained *Lactobacillus sporogenes* (1 × 10^8^ CFU/g)	Control bread	F/M	T2DM	52.0 ± 7.2	53.4 ± 7.6	8
Toejing et al. (61), Thailand	Parallel, R, DB	36	*Lactobacillus paracasei* HII01 (50 × 10^9^ CFU/d)	Placebo	F/M	T2DM	63.5 ± 5.94	61.78 ± 7.73	12
Ismail et al. (43) (A), Egypt	Parallel, R, DB	75	Probiotic yogurt containing *Bifidobacterium animalis dn-173010*	Balance diet	F/M	T2DM	48.3 ± 12.9	46.4 ± 13.2	16
Ismail et al. (43) (B), Egypt	Parallel, R, DB	75	Baking yeast daily containing *Saccharomyces cerevisiae*	Balance diet	F/M	T2DM	48.6 ± 11.5	46.4 ± 13.2	16
Tay et al., 2020 (60), New Zealand	Parallel, R, DB	26	*Lacticaseibacillus rhamnosus* (6 × 10^9^ CFU)	Placebo	F/M	T2DM	52.9 ± 8.7	54.1 ± 6.4	12
Kobyliak et al., 2018 (46), Ukraine	Parallel, R, DB	53	*Lactobacillus* + *Lactococcus* (6 × 10^10^ CFU/g), *Bifidobacterium* (1 × 10^10^ CFU/g), *Propionibacterium* (3 × 10^10^ CFU/g), *Acetobacter* (1 × 10^6^ CFU/g)	Placebo	F/M	T2DM	52.23 ± 1.74	57.18 ± 2.06	12
Hsieh et al. (42) (A), China	Parallel, R, DB	37	Live *Lactobacillus reuteri* ADR-1 (4 × 10^9^ CFU)	Placebo	F/M	T2DM	NR	NR	12
Hsieh et al. (42) (B), China	Parallel, R, DB	37	Heat-killed *Lactobacillus reuteri* ADR-3 (2 × 10^10^ CFU)	Placebo	F/M	T2DM	NR	NR	12
Rezaei et al. (55), Iran	Parallel, R, DB	90	2.5% fat probiotic yogurt (containing typical yogurt starter cultures, *plus Lactobacillus acidophilus* La5 and *Bifidobacterium lactis* Bb12)	Ordinary yogurt	F/M	T2DM	50.49 ± 10.92	50.13 ± 9.2	12

R, Randomized; DB, Double-blind; SB, Single-blind; T2DM, Type 2 Diabetes Mellitus; DN, Diabetic Nephropathy; GDM, Gestational Diabetes Mellitus; CFU, Colony Forming Units.

### 3.3 Quality of RCTs

In the risk of bias assessment, most studies had low risk regarding random sequence generation and allocation concealment. However, study by Ismail et al. (43) did not provide sufficient information on these aspects. Selective reporting posed a high risk in several studies. Only three studies did not follow double blinding procedure in their study protocol (37, 50, 57). Finally, most studies had low risk of incomplete outcome data (Table 2).

**Table 2:** Risk of bias assessment.

Studies	Random sequence generation	Allocation concealment	Selective reporting	Other sources of bias	Blinding (participants and personnel)	Blinding (outcome assessment)	Incomplete outcome data	General risk of bias
Toejing et al. (61)	L	L	H	U	L	U	L	H
Jamilian et al. (45)	L	L	H	U	L	U	L	H
Ismail et al. (43)	U	U	L	U	U	U	L	H
Tay et al. (60)	L	L	L	H	L	U	L	H
Mobini et al. (51)	L	L	L	U	L	U	L	M
Hajifaraji et al. (41)	L	L	L	U	L	U	L	M
Mazruei Arani et al. (49)	L	L	H	U	L	U	L	H
Jafarnejad et al. (44)	L	L	L	L	U	U	H	H
Sabico et al. (56)	L	L	L	L	L	U	L	L
Raygan et al. (54)	L	L	L	L	L	U	L	L
Kobyliak et al. (46)	L	L	L	U	L	U	L	M
Mafi et al. (47)	L	L	L	U	L	U	L	M
Hsieh et al. (42)	L	L	L	U	L	U	H	H
Firouzi et al. (40)	L	L	L	L	L	U	L	L
Miraghajani et al. (50)	L	L	L	L	H	U	L	H
Soleimani et al. (58)	L	L	L	U	L	U	L	M
Rezaei et al. (55)	L	L	H	U	L	U	L	H
Mohseni et al. (53)	L	L	L	U	L	U	L	M
Sato et al. (57)	L	L	H	U	H	H	L	H
Feizollahzadeh et al. (39)	L	L	H	L	L	U	H	H
Bayat et al. (37)	L	L	L	L	H	H	H	H
Bahmani et al. (36)	L	L	L	U	L	U	L	M
Badehnoosh et al. (35)	L	L	L	U	L	U	L	M
Babadi et al., (34)	L	L	H	U	L	U	L	H
Andreasen et al. (30)	L	L	H	L	L	U	L	H
Tonucci et al. (62)	L	L	L	U	L	U	H	H
Mohamadshahi et al. (52)	L	L	H	L	L	U	L	H
Asemi et al. (31)	L	L	L	L	L	U	L	L
Tajadadi-Ebrahimi et al. (59)	L	L	H	U	L	U	L	H
Asemi et al. (33)	L	L	L	L	L	U	L	L
Asemi et al. (32)	L	L	L	L	L	U	L	L
Mazloom et al. (48)	L	L	L	U	L	U	L	M
Ejtahed et al. (38)	L	L	H	L	L	U	L	H

L, Low, M, Moderate; H, High; U, Unclear.

### 3.4 Effect of probiotic on CRP

**Figure 2:**
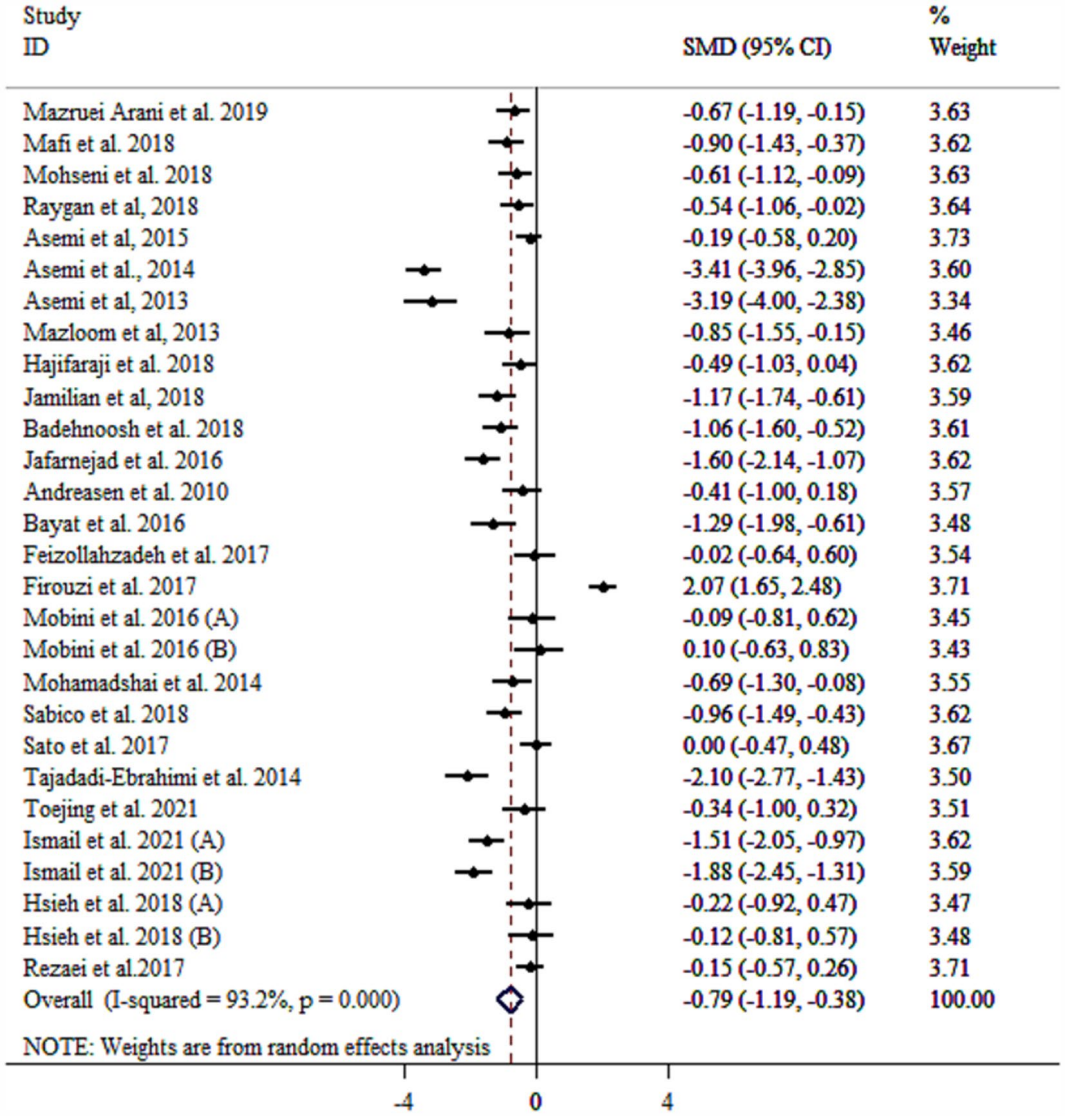
Forest plot detailing mean difference and 95% confidence intervals (CIs), the effects of probiotics supplementation on CRP.

Probiotic supplementation significantly reduced CRP levels in patients with diabetes (Figure 2). Substantial heterogeneity was observed. Several factors were identified as contributing to the high heterogeneity of the study, including age, sample size, health condition, and baseline BMI (Table 3). Subgroup analysis revealed the most substantial effects in patients with diabetic nephropathy, T2DM, as well as intervention duration ≥10 weeks, multi-strain probiotic, baseline BMI ≥30 kg/m^2^, and age < 55 years (Table 3). Sensitivity analysis confirmed the robustness of the overall findings (*p* < 0.05). Meta-regression analysis demonstrated no linear relationship between effect size and sample size or intervention duration (*p* > 0.05). Unlike Begg’s test (*p* > 0.05), there were significant small-study effects when performing Egger’s test (*p* = 0.033). According to the funnel plot, publication bias was evident (Supplementary Figure S2). Then, trim and fill analysis was performed with 34 studies (Six imputed studies, SMD = −1.09, 95% CI: −1.55, −0.64; *p* < 0.05).

**Table 3:** Subgroup analyses for the effects of probiotics supplementation on inflammation and oxidative stress biomarkers.

	Effect size, *n*	SMD (95% CI)	*I*^2^ (%)	P-heterogeneity
Probiotic on CRP				
Overall	28	−0.79 (−1.19, −0.38)	93.2	<0.001
Age (years)				
<55	16	−1.11 (−1.78, −0.45)	95.9	<0.001
≥55	10	−0.41 (−0.64, −0.18)	34.0	0.136
NR	2	−0.17 (−0.66, 0.32)	0.0	0.831
Sex				
Women	4	−1.08 (−1.54, −0.62)	64.6	0.037
Both	24	−0.74 (−1.21, −0.27)	93.9	<0.001
Study population				
Diabetic nephropathy	2	−0.78 (−1.15, −0.41)	0.0	0.532
Diabetic foot ulcer	1	−0.61 (−1.12, −0.09)	-	-
T2DM	21	−0.74 (−1.28, −0.20)	94.7	<0.001
GDM	4	−1.08 (−1.54, −0.62)	64.6	0.037
Intervention duration (week)				
<10	15	−0.39 (−0.91, 0.14)	92.7	<0.001
≥10	13	−1.25 (−1.82, −0.69)	92.0	<0.001
Sample size				
<60	14	−0.77 (−1.19, −0.34)	83.3	<0.001
≥60	14	−0.81 (−1.46, −0.15)	95.9	<0.001
Baseline BMI				
<30	17	−0.73 (−1.31, −0.15)		
≥30	9	−1.04 (−1.60, −0.48)	87.5	<0.001
NR	2	−0.17 (−0.66, 0.32)	0.0	0.530
Type of CRP				
Hs-CRP	21	−0.85 (−1.36, −0.33)	94.4	<0.001
CRP	7	−0.62 (−1.19, −0.05)	85.6	<0.001
Type of probiotic strains				
Single strains	15	−0.78 (−1.31, −0.26)	91.6	<0.001
Multi strains	13	−0.80 (−1.44, −0.15)	94.7	<0.001
Probiotic on IL-6				
Overall	15	−0.29 (−0.66, 0.09)	83.6	<0.001
Age (years)				
<55	10	−0.50 (−0.95, −0.05)	84.3	<0.001
≥55	3	0.17 (−0.75, 1.09)	84.7	<0.001
NR	2	0.16 (−0.33, 0.64)	0.0	0.588
Sex				
Women	2	−0.81 (−2.48, 0.87)	95.0	<0.001
Both	13	−0.20 (−0.57, 0.16)	79.6	<0.001
Study population				
T2DM	13	−0.20 (−0.57, 0.16)	79.6	<0.001
GDM	2	−0.81 (−2.48, 0.87)	95.0	<0.001
Intervention duration (week)				
<10	6	−0.15 (−0.88, 0.57)	89.1	<0.001
≥10	9	−0.37 (−0.80, 0.06)	79.6	<0.001
Sample size				
<60	10	−0.00 (−0.32, 0.32)	60.7	0.006
≥60	5	−0.80 (−1.51, −0.09)	89.6	<0.001
Baseline BMI				
<30	8	−0.20 (−0.74, 0.34)	85.9	<0.001
≥30	5	−0.59 (−1.25, 0.08)	85.3	<0.001
NR	2	0.16 (−0.33, 0.64)	0.0	0.588
Type of probiotic strains				
Single strains	9	−0.42 (−0.82, −0.01)	76.8	<0.001
Multi strains	6	−0.09 (−0.83, 0.65)	89.7	<0.001
Probiotic on TNF-a				
Overall	16	−1.35 (−2.05, −0.66)	94.2	<0.001
Age (years)				
<55	11	−1.35 (−2.08, −0.62)	93.3	<0.001
≥55	3	−6.62 (−11.20, −2.03)	97.9	<0.001
NR	2	−0.09 (−0.64, 0.46)	21.2	0.260
Sex				
Women	13	−0.86 (−1.54, −0.18)	92.8	<0.001
Both	3	−5.07 (−7.92, −2.22)	97.4	<0.001
Study population				
T2DM	13	−0.86 (−1.54, −0.18)	92.8	<0.001
GDM	3	−5.07 (−7.92, −2.22)	94.7	<0.001
Intervention duration (week)				
<10	7	−3.08 (−4.63, −1.52)	96.4	<0.001
≥10	9	−0.77 (−1.45, −0.09)	91.0	<0.001
Sample size				
<60	11	−1.74 (−2.80, −0.68)	95.1	<0.001
≥60	5	−1.16 (−1.92, −0.39)	90.6	<0.001
Baseline BMI				
<30	9	−2.22 (−3.38, −1.06)	95.7	<0.001
≥30	5	−1.03 (−2.04, −0.02)	92.8	<0.001
NR	2	−0.09 (−0.64, 0.46)	21.2	0.260
Type of probiotic strains				
Single strains	7	−1.67 (−2.96, −0.39)	95.4	<0.001
Multi strains	9	−1.15 (−1.98, −0.32)	93.3	<0.001
Probiotic on MDA				
Overall	14	−0.82 (−1.16, −0.47)	81.9	<0.001
Age (years)				
<55	8	−0.79 (−1.28, −0.30)	85.2	<0.001
≥55	6	−0.85 (−1.36, −0.35)	78.1	<0.001
Sex				
Women	10	−0.72 (−1.15, −0.28)	84.5	<0.001
Both	4	−1.06 (−1.54, −0.58)	64.6	0.037
Study population				
Diabetic nephropathy	2	−1.54 (−1.95, −1.13)	0.0	0.724
Diabetic foot ulcer	1	−0.75 (−1.27, −0.23)	-	-
T2DM	7	−0.47 (−0.96, 0.01)	81.9	<0.001
GDM	4	−1.06 (−1.54, −0.58)	64.6	0.037
Intervention duration (week)				
<10	4	−1.05 (−1.60, −0.50)	75.3	0.007
≥10	10	−0.72 (−1.15, −0.29)	83.3	<0.001
Sample size				
<60	7	−0.90 (−1.51, −0.29)	85.7	<0.001
≥60	7	−0.74 (−1.16, −0.32)	79.0	<0.001
Baseline BMI				
<30	9	−0.62 (−1.02, −0.21)	79.8	<0.001
≥30	5	−1.17 (−1.74, −0.60)	80.0	<0.001
Type of probiotic strains				
Single strains	5	−0.93 (−1.69, −0.18)	89.1	<0.001
Multi strains	9	−0.76 (−1.14, −0.37)	77.1	<0.001
Probiotic on GSH				
Overall	15	1.00 (0.41, 1.59)	94.2	<0.001
Age (years)				
<55	10	1.02 (0.19, 1.85)	95.7	<0.001
≥55	5	0.94 (0.22, 1.66)	87.5	<0.001
Sex				
Women	11	1.27 (0.47, 2.07)	95.6	<0.001
Both	4	0.30 (−0.04, 0.64)	38.0	0.184
Study population				
Diabetic nephropathy	2	0.68 (0.09, 1.28)	60.9	0.110
Diabetic foot ulcer	1	0.16 (−0.34, 0.67)	-	-
T2DM	8	1.57 (0.45, 2.70)	96.8	<0.001
GDM	4	0.30 (−0.04, 0.64)	38.0	0.184
Intervention duration (week)				
<10	4	0.51 (0.17, 0.84)	41.3	0.164
≥10	11	1.19 (0.37, 2.01)	95.7	<0.001
Sample size				
<60	7	0.95 (0.10, 1.80)	92.8	<0.001
≥60	8	1.04 (0.18, 1.90)	95.5	<0.001
Baseline BMI				
<30	9	1.15 (0.25, 2.04)	95.9	<0.001
≥30	6	0.78 (0.07, 1.48)	89.6	<0.001
Type of probiotic strains				
Single strains	5	1.82 (0.16, 3.49)	97.7	<0.001
Multi strains	10	0.59 (0.15, 1.03)	84.4	<0.001
Probiotic on NO				
Overall	10	0.60 (0.30, 0.91)	72.0	<0.001
Age (years)				
<55	6	0.61 (0.28, 0.95)	60.0	0.028
≥55	4	0.59 (−0.05, 1.23)	83.0	<0.001
Sex				
Women	3	0.35 (−0.03, 0.72)	32.3	0.228
Both	7	0.71 (0.32, 1.10)	74.9	<0.001
Study population				
Diabetic nephropathy	2	0.11 (−0.64, 0.87)	76.8	0.038
Diabetic foot ulcer	1	0.93 (0.39, 1.46)	-	-
T2DM	4	0.94 (0.56, 1.32)	53.2	0.093
GDM	3	0.35 (−0.03, 0.72)	32.3	0.228
Intervention duration (week)				
<10	4	0.59 (−0.05, 1.23)	83.0	<0.001
≥10	6	0.61 (0.28, 0.95)	60.0	0.028
Sample size				
<60	4	0.75 (0.31, 1.19)	59.0	0.062
≥60	6	0.51 (0.09, 0.93)	76.5	<0.001
Baseline BMI				
<30	6	0.58 (0.35, 0.81)	19.0	0.290
≥30	4	0.65 (−0.12, 1.42)	87.5	<0.001
Type of probiotic strains				
Single strains	3	0.58 (−0.27, 1.43)	88.7	<0.001
Multi strains	7	0.62 (0.33, 0.91)	51.4	0.055
Probiotic on TAC				
Overall	16	0.48 (0.27, 0.69)	61.7	<0.001
Age (years)				
<55	11	0.36 (0.16, 0.57)	45.1	0.051
≥55	5	0.76 (0.28, 1.23)	72.6	0.006
**Sex**				
Women	4	0.68 (0.40, 0.96)	2.7	0.379
Both	12	0.42 (0.17, 0.67)	66.0	<0.001
Study population				
Diabetic nephropathy	2	0.35 (−0.01, 0.71)	0.0	0.760
Diabetic foot ulcer	1	1.66 (1.07, 2.25)	-	-
T2DM	9	0.30 (0.09, 0.51)	37.1	0.122
GDM	4	0.68 (0.40, 0.96)	2.7	0.379
Intervention duration (week)				
<10	4	0.72 (0.15, 1.30)	78.7	0.003
≥10	12	0.40 (0.20, 0.61)	48.0	0.032
Sample size				
<60	8	0.42 (0.14, 0.70)	49.4	0.054
≥60	8	0.55 (0.22, 0.87)	72.1	<0.001
Baseline BMI				
<30	10	0.57 (0.27, 0.86)	69.7	<0.001
≥30	6	0.35 (0.07, 0.63)	40.4	0.136
Type of probiotic strains				
Single strains	6	0.30 (0.08, 0.52)	24.0	0.254
Multi strains	10	0.58 (0.28, 0.88)	67.6	<0.001

### 3.5 Effect of probiotic on IL-6

**Figure 3:**
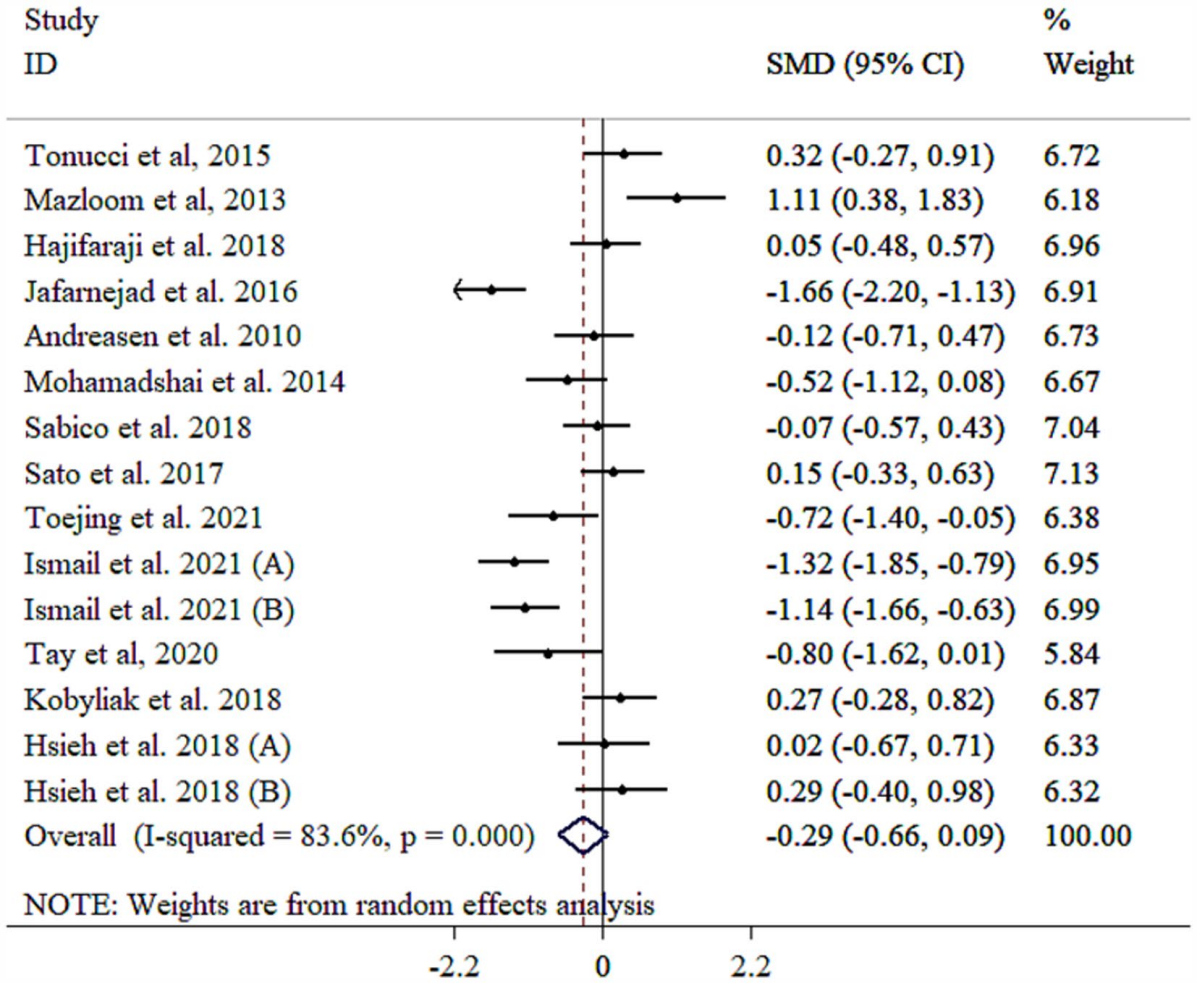
Forest plot detailing mean difference and 95% confidence intervals (CIs), the effects of probiotics supplementation on IL-6.

IL-6 level did not significantly decrease following probiotic supplementation with substantial heterogeneity (Figure 3). Subgrouping by gender, sample size, and duration reduced heterogeneity between studies (Table 3). However, removing the study by Mazloom et al. (48), using sensitivity analysis made the overall results statistically significant (SMD = −0.38, 95% CI: −0.74, −0.02; *p* < 0.05). Subgroup analyses showed significant decreases in IL-6 in a sample size of ≥60 with mean age of <50 years (p < 0.05), as well as studies administered single-strain probiotic (Table 3). Meta-regression analysis showed that effect size did not have a linear relationship with sample size and intervention duration (*p* > 0.05). Non-significant outcomes from Egger’s and Begg’s tests validate the reliability of the meta-analysis results (*p* > 0.05). A visual inspection of the funnel plot revealed that the distribution of studies was symmetrical (Supplementary Figure S3).

### 3.6 Effect of probiotic on TNF-a

**Figure 4:**
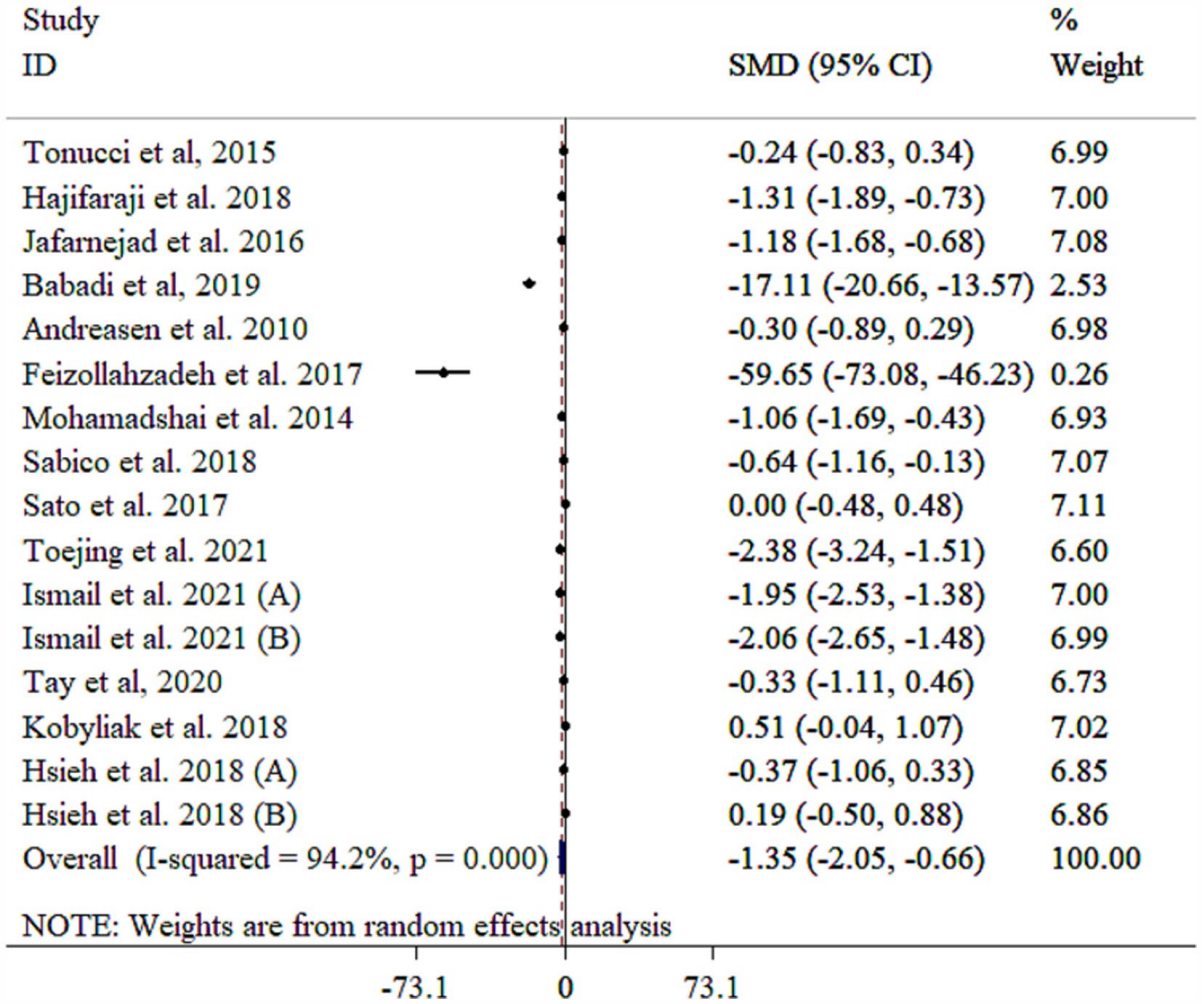
Forest plot detailing mean difference and 95% confidence intervals (CIs), the effects of probiotics supplementation on TNF-a.

Probiotic supplements significantly reduced TNF-*α* (Figure 4). Heterogeneity among the included studies was high that was reduced following subgroup analysis based on mean age (Table 3). Subgroup analysis revealed the most substantial effects in patients with diabetic nephropathy, intervention duration <10 weeks, single-strain probiotic, baseline BMI <30 kg/m^2^, sample size of <60, and mean age of ≥55 years (Table 3). Sensitivity analysis confirmed that the pooled results were stable and not influenced by any single study (*p* < 0.05). Meta-regression analysis revealed that effect size did not have a linear relationship with sample size and intervention duration (*p* > 0.05). Egger’s test, unlike Begg’s test (*p* > 0.05), indicated evidence of publication bias (*p* = 0.001). Publication bias was also detected through visual inspection of the funnel plot (Supplementary Figure S4). Nevertheless, the results remained significant after conducting a trim and fill analysis with 20 studies (Four imputed studies, SMD = −2.19, 95% CI: −3.05, −1.34; *p* < 0.05).

### 3.7 Effect of probiotic on MDA

**Figure 5:**
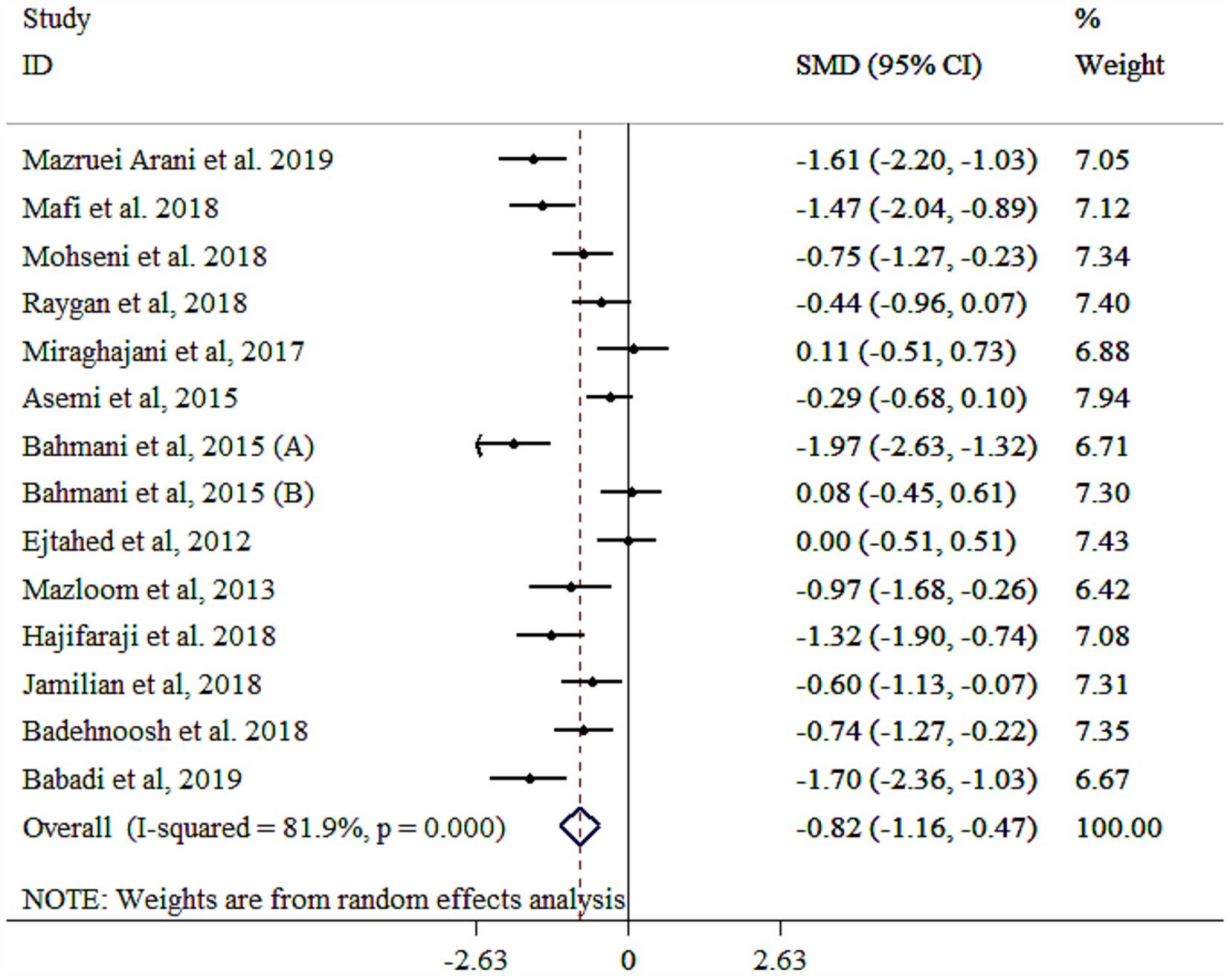
Forest plot detailing mean difference and 95% confidence intervals (CIs), the effects of probiotics supplementation on MDA.

The analysis revealed that probiotics significantly reduced MDA levels, although with substantial heterogeneity (Figure 5). A subgroup analysis identified the study population as the primary source of this heterogeneity (Table 3). The subgroup analysis indicated consistent effects across various populations and study protocols, with the largest reductions observed in single-strain probiotics, diabetic nephropathy patients, both genders, those aged ≥55 years, interventions lasting <10 weeks, studies with a sample size <60, and individuals with a baseline BMI ≥30 kg/m^2^ (Table 3). Sensitivity analysis confirmed the robustness of these findings (*p* < 0.05). Meta-regression analysis showed no significant influence of sample size or intervention duration on effect size (*p* > 0.05). While Egger’s (*p* = 0.038) and Begg’s (*p* = 0.025) tests indicated potential publication bias, the funnel plot (Supplementary Figure S5) displayed a symmetric distribution, suggesting no significant bias.

### 3.8 Effect of probiotic on GSH

**Figure 6:**
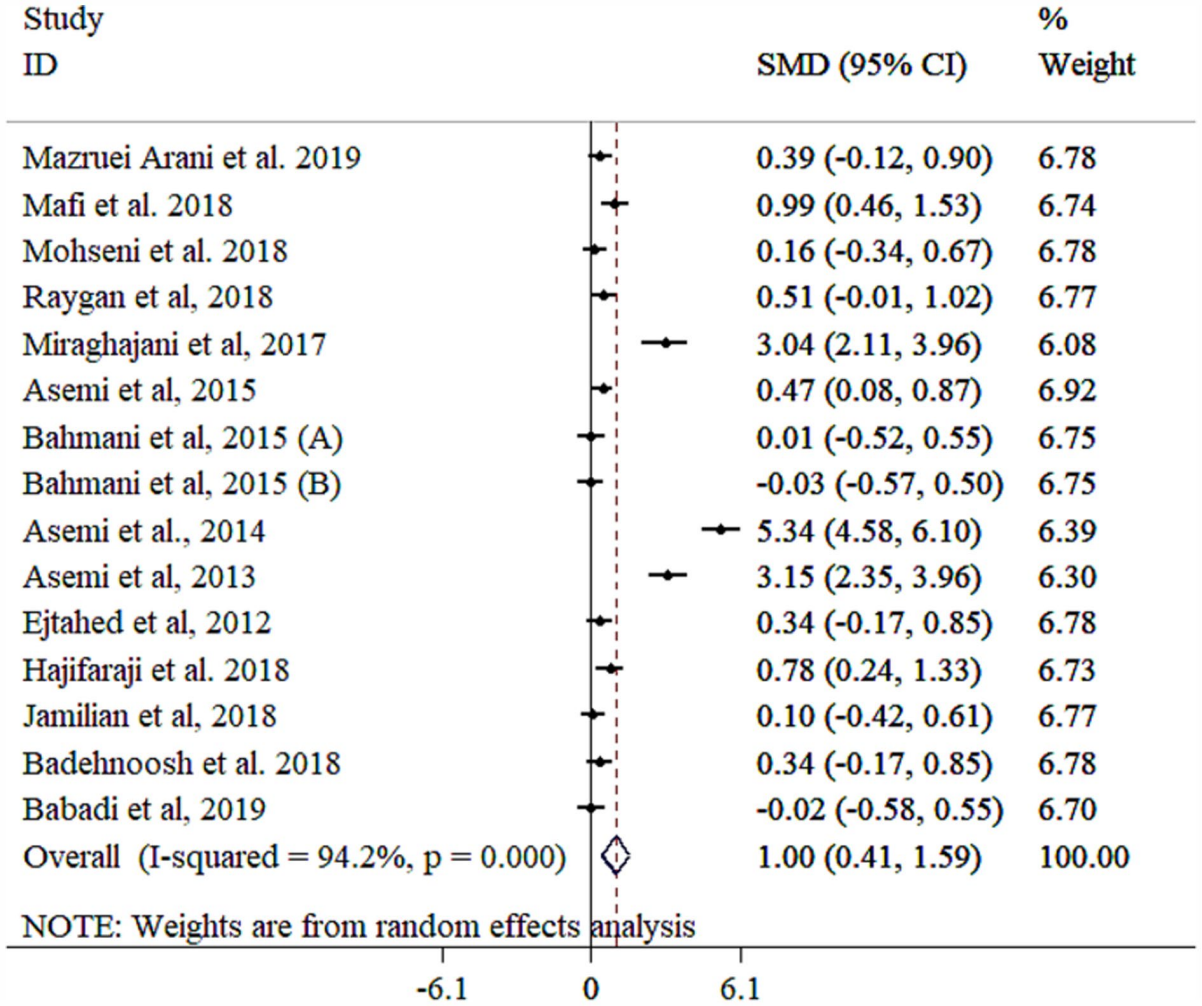
Forest plot detailing mean difference and 95% confidence intervals (CIs), the effects of probiotics supplementation on GSH.

A significant increase in GSH levels was observed following probiotic supplementation (Figure 6), although high between-study heterogeneity was observed. Subgroup analyses reduced heterogeneity when stratified by gender, study population, and intervention duration (Table 3). Greater effects were noted in studies with longer interventions (≥10 weeks), single-strain probiotics, higher sample sizes (≥60), baseline BMI <30 kg/m^2^, participants with T2DM, mean age < 55 years, and female participants (Table 3). Sensitivity analysis confirmed the reliability of the findings (*p* < 0.05). Meta-regression did not identify significant moderators of effect size (*p* > 0.05). While Egger’s test (*p* = 0.006) indicated potential publication bias, Begg’s test did not detect bias (*p* > 0.05). Funnel plot analysis revealed asymmetry (Supplementary Figure S6). However, the trim-and-fill method validated the significant effect of probiotics on GSH levels with 19 studies (Four imputed studies, SMD = 1.22, 95% CI: 0.76, 1.44; *p* < 0.05).

### 3.9 Effect of probiotic on NO

**Figure 7:**
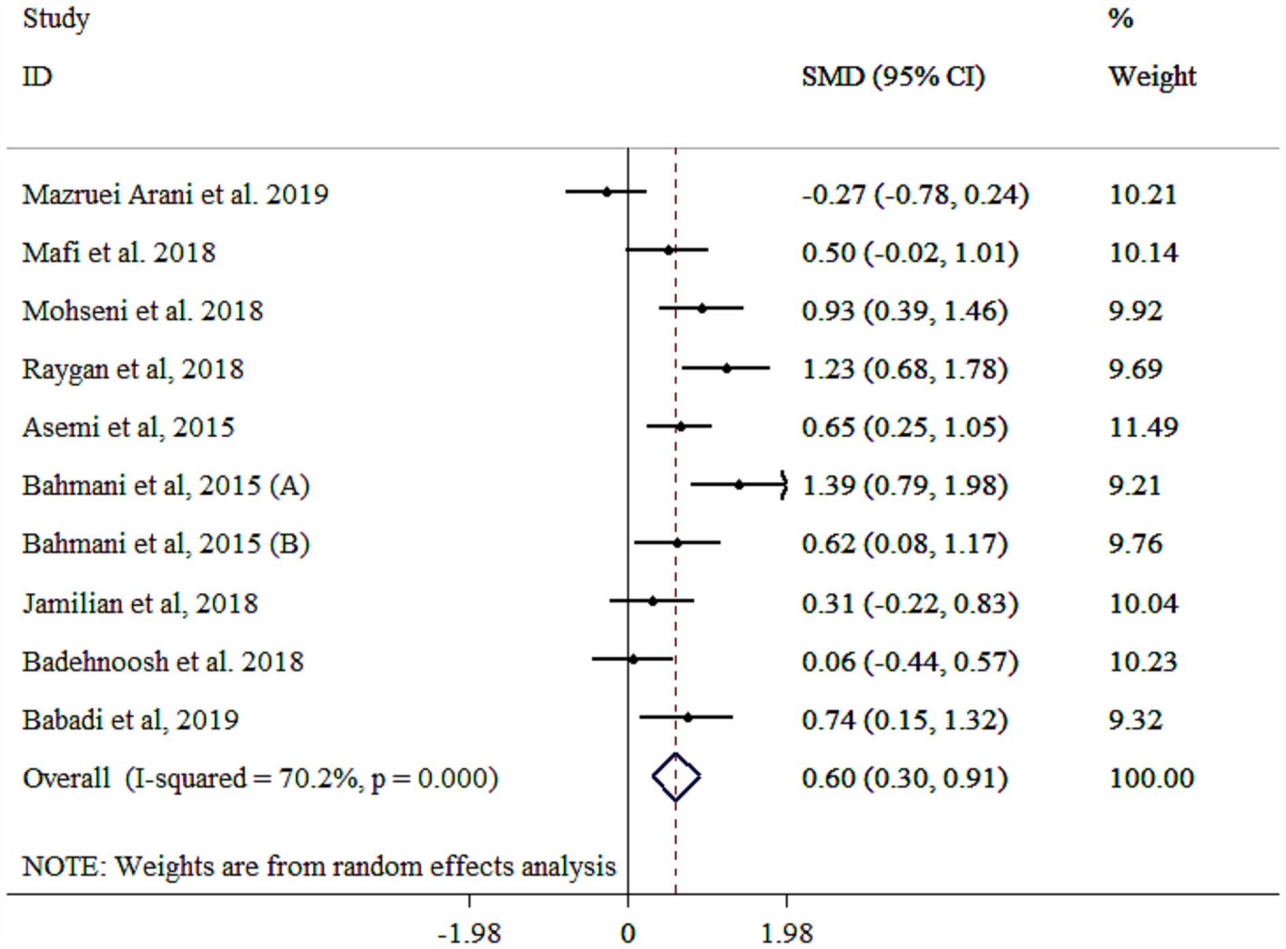
Forest plot detailing mean difference and 95% confidence intervals (CIs), the effects of probiotics supplementation on NO.

Probiotic supplementation significantly increased NO levels, though substantial heterogeneity was observed (Figure 7). Subgroup analyses identified gender, study population, sample size, and baseline BMI as key contributors to this heterogeneity (Table 3). Larger effects were observed in younger participants (<55 years), multi-strain probiotics, longer interventions (≥10 weeks), smaller sample sizes (<60), individuals with T2DM, baseline BMI <30 kg/m^2^, and both genders (Table 3). The robustness of findings was approved by sensitivity analysis (*p* < 0.05). Moreover, sample size and study duration did not influence the final results according to meta-regression analysis (*p* > 0.05). Publication bias assessments provided mixed results, with Egger’s test showing no bias (*p* > 0.05) and Begg’s test indicating potential bias (*p* = 0.025). A moderate asymmetry was detected in the funnel plot (Supplementary Figure S7). However, trim-and-fill analysis yielded an adjusted significant effect with 11 studies (One imputed study, SMD = 0.52, 95% CI: 0.21, 0.84; *p* < 0.05).

### 3.10 Effect of probiotic on TAC

**Figure 8:**
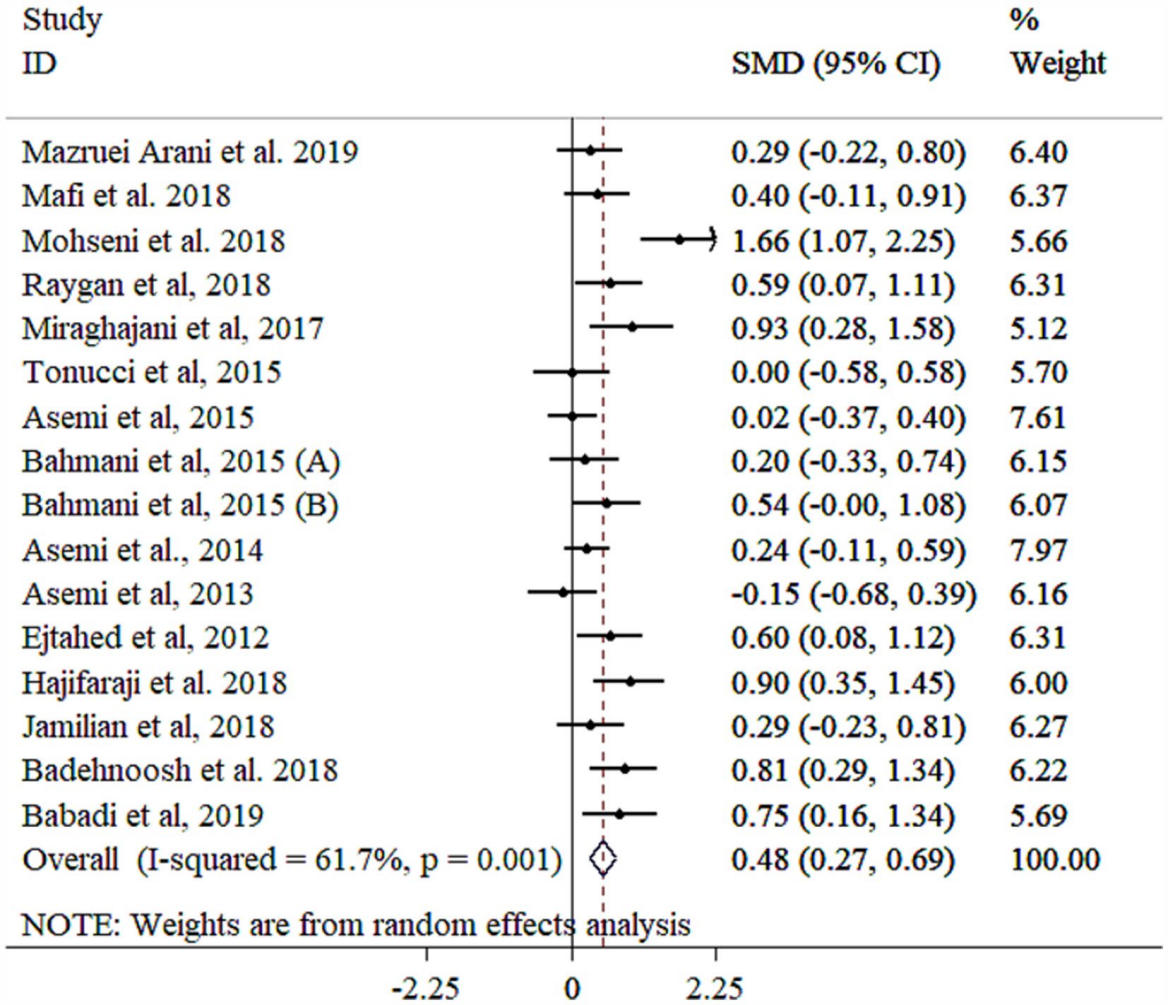
Forest plot detailing mean difference and 95% confidence intervals (CIs), the effects of probiotics supplementation on TAC.

Probiotic supplementation significantly improved TAC levels (Figure 8), with significant heterogeneity across studies. Subgroup analyses identified age, gender, study population, BMI, and sample size as primary sources of heterogeneity (Table 3). Greater effects were observed in female participants, those with BMI <30 kg/m^2^, mean age ≥ 55 years, and individuals with GDM, particularly in studies using multi-strain probiotics, larger sample sizes (≥60), and shorter intervention durations (<10 weeks) (Table 3). Sensitivity analysis confirmed the robustness of the findings (*p* < 0.05). Meta-regression analysis showed no significant linear association between effect size and sample size or study duration (*p* > 0.05). While Egger’s (*p* = 0.043) and Begg’s (*p* = 0.019) tests suggested potential publication bias, the funnel plot displayed no evidence of asymmetry (Supplementary Figure S8).

### 3.11 GRADE assessment of evidence

Table 4 summarizes the findings of the meta-analysis and evaluates the quality of evidence using the GRADE framework for the effect of probiotic supplementation on oxidative stress and inflammatory biomarkers in patients with diabetes. Evidence was frequently downgraded due to serious risk of bias and imprecision, while inconsistency, indirectness, and publication bias were generally not major concerns. Moderate-quality evidence supported significant effects on MDA, GSH, and NO, while low-quality evidence was found for CRP, TNF-*α*, IL-6, and TAC.

**Table 4:** Summary of findings and quality of evidence the probiotics supplementation on oxidative stress and inflammatory biomarkers.

Outcome measures	Summary of findings		Quality of evidence assessment (GRADE)					
	No of patients (effect size)	Effect size (95% CI)	Risk of bias	Inconsistency	Indirectness	Imprecision	Publication bias	Quality of evidence
SMD analysis								
CRP	1,696 (28)	−0.79 (−1.19, −0.38)	Serious	Not Serious	Not Serious	Serious	Not Serious	Low
TNF-α	818 (16)	−1.35 (−2.05, −0.66)	Serious	Not Serious	Not Serious	Serious	Not Serious	Low
IL-6	812 (16)	−0.29 (−0.66, 0.09)	Serious	Not Serious	Not Serious	Serious	Not Serious	Low
MDA	805 (14)	−0.82 (−1.16, −0.47)	Serious	Not Serious	Not Serious	Not Serious	Not Serious	Moderate
TAC	994 (16)	0.48 (0.27, 0.69)	Serious	Not Serious	Not Serious	Serious	Not Serious	Low
GSH	949 (15)	1.00 (0.41, 1.59)	Serious	Not Serious	Not Serious	Not Serious	Not Serious	Moderate
NO	615 (10)	0.60 (0.30, 0.91)	Serious	Not Serious	Not Serious	Not Serious	Not Serious	Moderate

CRP, C-reactive protein; TNF, tumor necrosis factor; IL-6, interleukin-6; TAC, total antioxidant capacity; MDA, Malondialdehyde; GSH, Glutathione; NO, Nitric oxide.

## 4 Discussion

This study comprehensively evaluated the effects of probiotics on inflammatory and oxidative stress biomarkers in diabetic populations by summarizing the results of previous meta-analysis and systematic review studies, as well as performing an updated meta-analysis on RCTs. Meta-research of meta-analysis studies revealed that probiotics, particularly strains like *Lactobacillus*, *Bifidobacterium*, and *Streptococcus*, emerged as promising interventions for reducing inflammation and oxidative stress, as evidenced by the significant reductions in markers like CRP, TNF-α, and MDA, alongside improvements in GSH and TAC levels. However, the effects on NO and IL-6 were inconsistent, with several studies reporting no significant changes.

Our meta-analysis results also demonstrated that probiotics had a significant improving effect on inflammatory and oxidative stress markers. However, contrary to most previous meta-analyses, our study revealed a significant increase in NO following probiotic supplementation, which was further confirmed through sensitivity analysis. Consistent with the majority of prior studies, our meta-analysis did not report a significant effect on IL-6. Nonetheless, sensitivity analysis indicated that probiotics could significantly reduce IL-6 levels. In all our pooled analyses, high heterogeneity, stemming from differences in methodology, various probiotic strains, and diverse study populations, reduced the certainty of the findings. Our result must be interpreted with caution due to high heterogeneity. Although subgroup analysis and meta-regression were used to identify the factors contributing to heterogeneity, the low methodological quality of most RCTs conducted so far underscores the need for higher-quality studies with larger sample sizes to enable definitive conclusions in this area.

The conflicting results of meta-analysis studies can be attributed to various factors. First, none of the meta-analysis studies were as comprehensive as ours and had missed some articles. Additionally, certain methodological flaws in these studies could have influenced their results. Some studies used WMD analysis (9, 12–14, 21–23, 25–29). Since various kits and methods with differing sensitivities have been used for measuring biochemical factors, failing to standardize the effect size based on the standard deviation and reporting raw mean differences cannot provide an accurate estimate of the impact of probiotics on biochemical markers (63, 64).

Regarding the subgroup analysis results, although both age groups benefited from probiotic supplementation in improving inflammation and antioxidant status, the effects appear to be more pronounced in individuals under 55 years of age. The observed greater efficacy of probiotic supplementation in individuals under 55 years of age, compared to older adults, may be attributed to several factors. Younger individuals typically have a more diverse and resilient gut microbiota, which can enhance the colonization and activity of administered probiotics, leading to more pronounced anti-inflammatory and antioxidant effects. In contrast, aging is associated with a gradual decline in immune function which may reduce the responsiveness of body to probiotics. Additionally, older adults often experience a natural decrease in gut microbiota diversity and stability, potentially diminishing the effectiveness of probiotic interventions (65). Therefore, the age-related differences in gut microbiota composition and immune system functionality likely contribute to the enhanced benefits of probiotics observed in the younger population. Regarding gender, the effects of probiotics do not appear to be dependent on sex, as both males and females benefit from the positive impacts of probiotics. However, a previous review highlighted differences in the responses of women and men to probiotics, possibly due to variations may be linked to differences in gut microbiota composition (66). Regarding study population, the largest reductions were observed in populations with diabetes-related conditions, such as GDM and diabetic nephropathy. These populations typically exhibit higher baseline levels of inflammation (67, 68), providing a greater scope for improvement. Regarding the duration of supplementation, probiotics did not show significant effects on certain biomarkers, including NO, IL-6, and CRP, in short-term interventions (<10 weeks). However, long-term supplementation (≥10 weeks) significantly improved all markers except IL-6. This finding suggests that probiotics are more effective with prolonged supplementation. Due to the diversity of probiotics and the varying doses studied, subgroup analysis based on these factors was not feasible. However, subgroup analysis based on single and multi-strain probiotics showed that multi-strain probiotic supplements do not necessarily have more pronounced beneficial effects than single-strain ones. Both types of supplements can significantly improve inflammation and oxidative stress, consistent with previous findings (69).

The molecular mechanisms by which probiotics exert their effects on inflammatory and oxidative stress markers are multifaceted and increasingly well-understood. Probiotics modulate systemic inflammation primarily through their influence on the gut microbiota, restoring dysbiosis and strengthening the intestinal epithelial barrier (70). This prevents translocation of lipopolysaccharide (LPS) from gram-negative bacteria, thereby reducing activation of Toll-like receptor 4 (TLR4) and downstream nuclear factor-kappa-B (NF-κB) signaling, a key driver of pro-inflammatory cytokine production such as CRP, TNF-*α*, and IL-6 (71–73). Novel insights suggest that probiotics also induce epigenetic modifications, including histone deacetylation and microRNA regulation, to suppress the transcription of pro-inflammatory genes (74). Moreover, probiotics have also been shown to regulate the NLRP3 inflammasome, which plays a pivotal role in the activation of caspase-1 and the release of interleukin-1-beta (IL-1β) (75). When IL-1β binds to its receptor, it activates intracellular signaling pathways, like the NF-κB pathway (76). In oxidative stress regulation, probiotics enhance the antioxidant defense systems by modulating redox-sensitive signaling pathways. For instance, they activate nuclear factor erythroid 2-related factor 2 (Nrf2), a master regulator of antioxidant gene expression, leading to an increase in TAC (77, 78). Recent studies highlight that certain probiotic strains produce bioactive metabolites, such as exopolysaccharides and indole derivatives, which directly scavenge reactive oxygen species (ROS) and mitigate lipid peroxidation, thereby lowering MDA levels (79, 80). Moreover, probiotics have been shown to modulate NO metabolism by influencing endothelial nitric oxide synthase (eNOS) activity (81). This novel mechanism involves increasing the availability of arginine, the substrate for NO synthesis (82), while concurrently reducing asymmetric dimethylarginine (ADMA), an eNOS inhibitor (83). These emerging molecular insights underscore the multifaceted and innovative roles of probiotics in reducing inflammation and oxidative stress, highlighting their therapeutic potential in managing chronic conditions characterized by these pathological processes.

In terms of clinical mechanisms, reducing inflammatory markers such as CRP and TNF-*α* can significantly alleviate systemic inflammation, thereby decreasing the risk of complications like vascular dysfunction and insulin resistance (84, 85). Lower levels of MDA, a marker of oxidative stress, indicate reduced cellular and tissue damage, particularly to endothelial cells and blood vessels, which are often impaired in diabetic and cardiovascular conditions (86). Increasing GSH and TAC enhances the antioxidant defenses of body, helping to neutralize free radicals and mitigate oxidative stress, thereby preserving cellular integrity and improving endothelial function (87). Elevated NO levels promote vasodilation, improving blood flow and reducing vascular resistance, which can lower blood pressure and reduce cardiovascular strain (88). Moderate-quality evidence supported significant effects on MDA, GSH, and NO, while low-quality evidence was found for CRP, TNF-α, IL-6, and TAC. Therefore, probiotics cannot yet be definitively recommended as a therapeutic approach for improving inflammation and oxidative stress in diabetic patients, and higher-quality studies need to be conducted.

This meta-analysis possesses several strengths, including rigorous methodology and the application of a various statistical analyses to capture a valid result. Consistent findings across diverse populations further reinforce the reliability of the results. However, some limitations warrant attention. First, subgroup analysis could not be performed on probiotic type and dosage due to variations in administered interventions. Therefore, strain-specific effects of probiotic on each biomarker were not be investigated. However, we performed subgroup analysis based on multi strain/single strain. Second, the high heterogeneity in most of analyses decreased the validity of findings. However, we tried to investigate the possible sources of it by performing subgroup analysis and meta-regression. Third, most included studies were short-term, limiting insights into the sustained effects of probiotics on inflammation and oxidative stress markers. However, subgroup analysis and meta-regression based on intervention duration could determine the impact of duration on effect sizes. Fourth, most of the RCTs conducted to date have methodological limitations, resulting in lower certainty of evidence. Therefore, additional high-quality studies are needed in this field to strengthen the findings and improve the reliability of the conclusions. Additionally, future studies should focus on strain-specific effects of probiotics on each inflammatory and oxidative stress biomarkers. This approach would provide more precise insights into how different probiotic strains contribute to modulating inflammation and oxidative stress in various populations, ultimately enhancing personalized therapeutic strategies.

## 5 Conclusion

This meta-analysis highlights the significant potential of probiotics in improving inflammatory and oxidative stress markers like CRP, TNF-*α*, and MDA, while boosting antioxidant defenses such as GSH, TAC, and NO in patients with diabetes mellitus. However, the non-significant effect on IL-6 suggests variability in strain-specific actions. Moderate-quality evidence supported significant effects on MDA, GSH, and NO, while low-quality evidence was found for CRP, TNF-α, IL-6, and TAC. Therefore, probiotics cannot yet be definitively recommended as a therapeutic approach for improving inflammation and oxidative stress in diabetic patients, and higher-quality studies with a strain-specific approach need to be conducted.

## Data Availability

The datasets presented in this study can be found in online repositories. The names of the repository/repositories and accession number(s) can be found in the article/Supplementary material.
