# Supplementary material for: The impact of probiotics on oxidative stress and inflammatory markers in patients with diabetes: a meta-research of meta-analysis studies
Source: Front Nutr. 2025 Mar 7;12:1552358. doi: 10.3389/fnut.2025.1552358 (PMC11926743; doi:10.3389/fnut.2025.1552358)
Supplement: Supplementary file 1 [file Data_Sheet_1.docx]

**Supplementary files:**

Records excluded (n=3)

-did -Did not provide information's (n=3)

- GG

-Other irrelevant studies (2)

- Non appropriate design (1)

-Other irrelevant studies (2)

- Non appropriate design (1)

Records excluded (n=597)

-Unrelated title and abstract (n=384)

-Animal studies (n=216)

**Fig. 1.** PRISMA Flow diagram.

Records screened by title/abstract

(n=615)

Duplicates excluded

(n=94)

Included studies

(n=15)

Full-text articles evaluated for eligibility

(n=18)

Total records identified through database searching

(n=709)

**
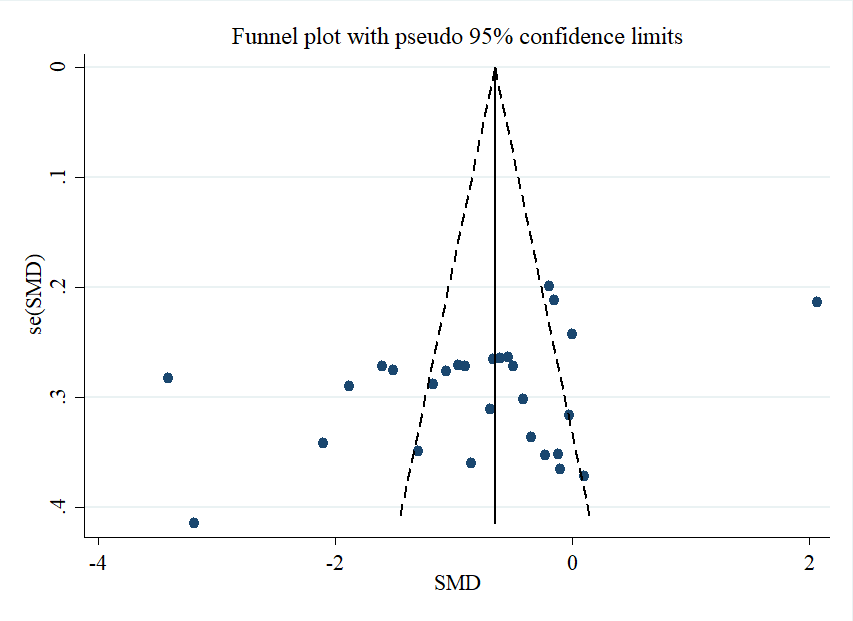
**

**Fig. 2.** CRP

**
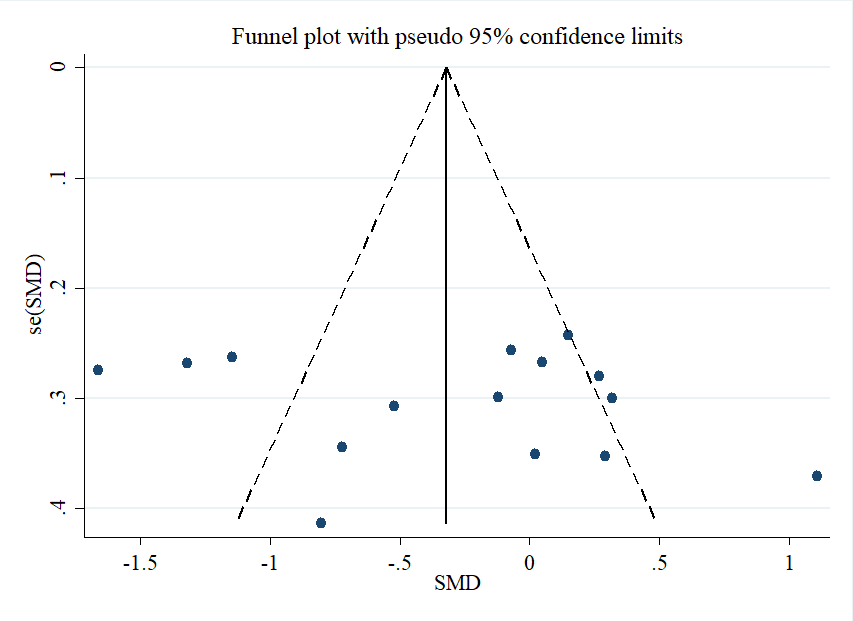
**

**Fig. 3.** IL-6

**
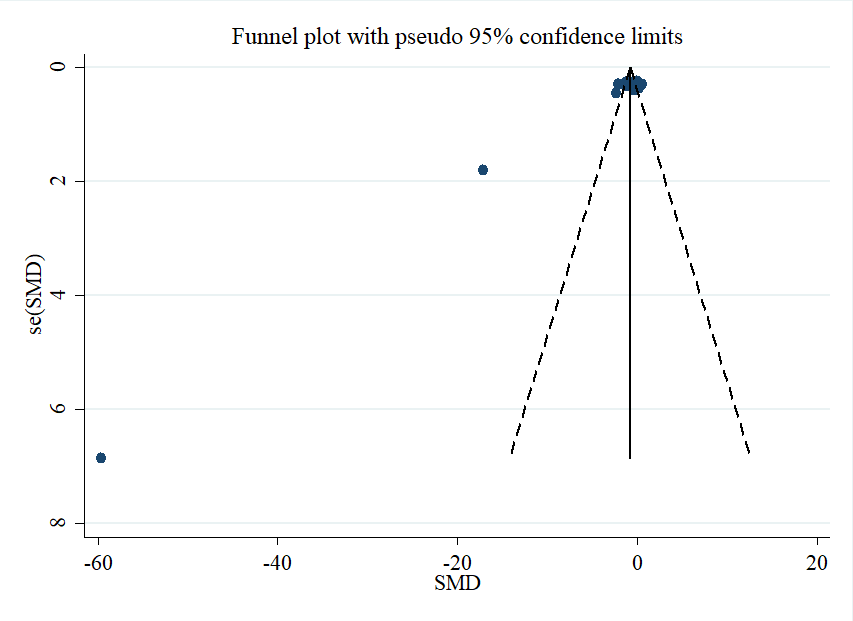
**

**Fig. 4.** TNF-a

**
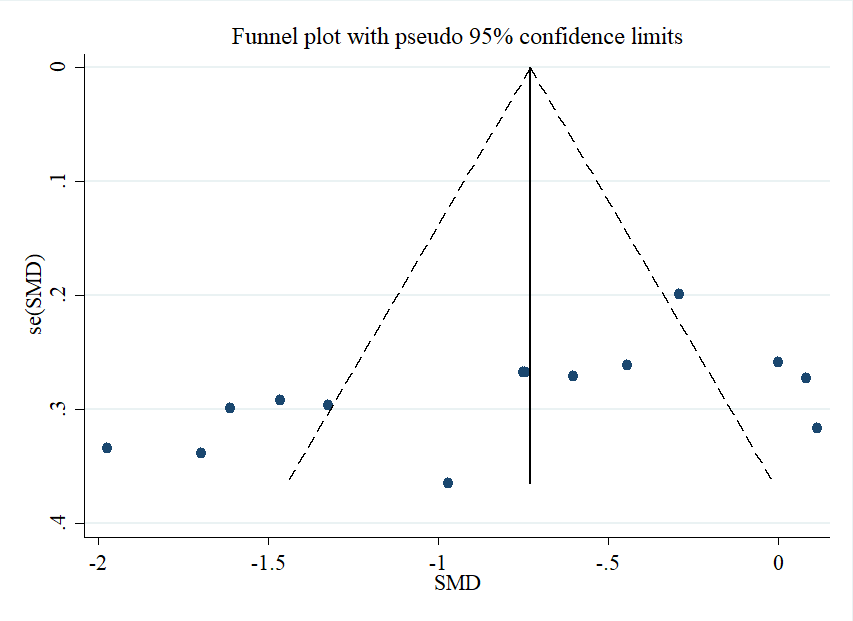
**

**Fig. 5.** MDA.

**
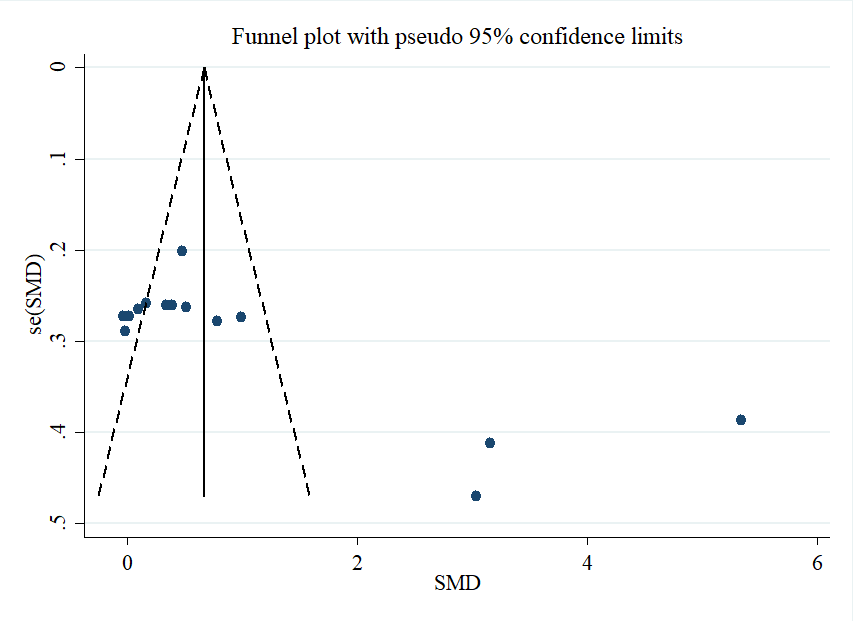
**

**Fig. 6.** GSH

**
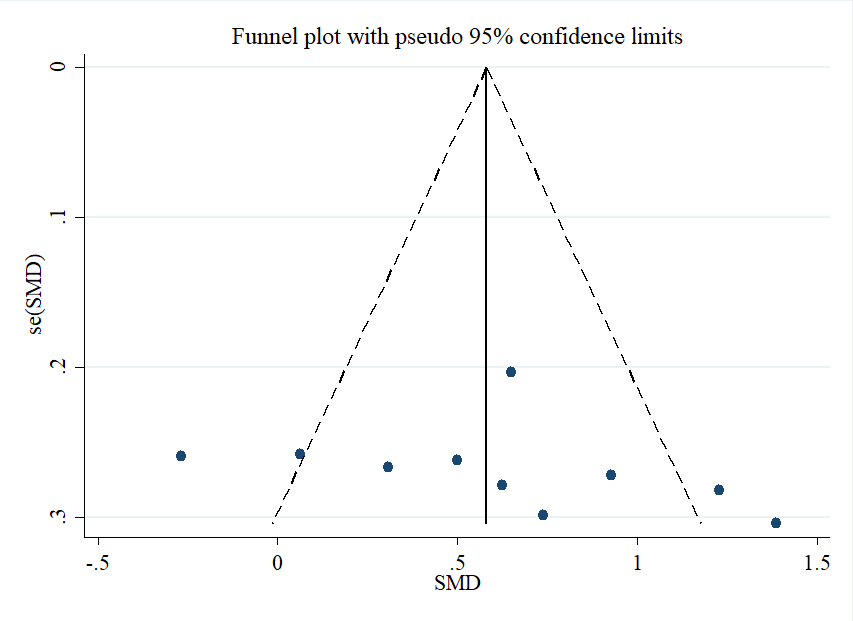
**

**Fig. 7.** NO

**
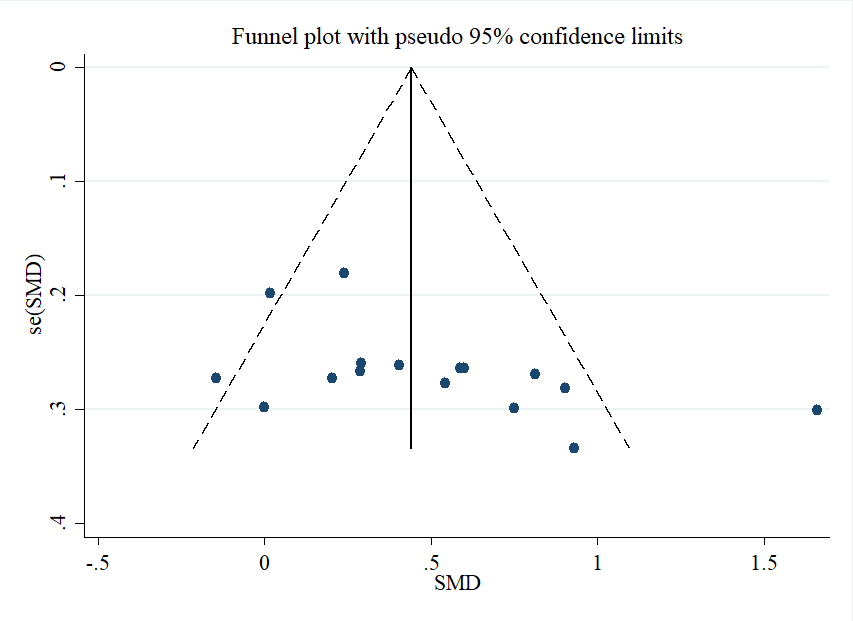
**

**Fig. 8.** TAC

**Table S1:** Study characteristics of included meta-analysis studies

| **Citation (First author et al., year)** | **No. of Studies** | **Location** | **Sample Size and Study Population** | **Age(year)** | **Intervention** | **Quality Assessment** | **Effect Size** | **Main Finding(s)** |
| --- | --- | --- | --- | --- | --- | --- | --- | --- |
| Kocsis et al.2020 [1] | 16 | Hungary | 937 – T2DM | NR | *Lactobacillus, Bifidobacterium, Streptococcus, Saccharomyces* | Yes (Cochrane)  15/16 high | WMD | ↓ CRP |
| Yao et al. 2017 [2] | 4 | China | 257 – T2DM | 54 | *Lactobacillus, Bifidobacterium* | Yes (Cochrane)  0/4 high | WMD | ↓ CRP |
| Tabrizi et al. 2019 [3] | 13 | Iran | 740 - Diabetes | 46 | *Lactobacillus, Bifidobacterium, Streptococcus* | Yes (Cochrane)  13/13 high | SMD | ↑ NO  ↓ TNF-α, CRP  ↔ IL-6 |
| Hasain et al. 2021 [4] | 4 | Malaysia | 221 - GDM | 29 | *Lactobacillus, Bifidobacterium, Streptococcus* | Yes (Cochrane)  4/4 high | WMD | ↓ TNF-α, CRP, IL-6, MDA  ↔ NO, TAC, GSH |
| Zheng et al. 2019 [5] | 3 | China | 168 - GDM | 30 | *Lactobacillus, Bifidobacterium, Streptococcus* | Yes (Cochrane)  3/3 high | WMD | ↑ NO, TAC, GSH  ↓ MDA |
| Ardeshirlarijan et al. 2019 [6] | 13 | Iran | 809 – T2DM | 56 | *Lactobacillus, Bifidobacterium, Streptococcus* | Yes (Jadad)  13/13 high | SMD | ↑ TAC, GSH  ↓ MDA  ↔ NO |
| Samah et al. 2016 [7] | 3 | Malaysia | 193 – T2DM | NR | *Lactobacillus, Bifidobacterium, Streptococcus* | Yes (Cochrane)  0/3 high | WMD | ↔ IL-6, CRP, MDA |
| Chan et al. 2021 [8] | 7 | China | 248 - GDM | NR | NR | Yes (Cochrane)  6/7 high | WMD | ↑ GSH  ↓ MDA, CRP, IL-6  ↔ NO, TAC, TNF-α |
| Kasińska et al. 2015 [9] | 4 | Poland | 438 – T2DM | NR | *Lactobacillus, Bifidobacterium* | Yes (Jadad)  4/4 high | SMD | ↔ CRP |
| Chen et al. 2020 [10] | 7 | China | 245 - GDM | 29 | *Lactobacillus, Bifidobacterium* | Yes (Jadad)  7/7 high | WMD | ↑ NO  ↓ MDA, CRP  ↔ GSH |
| Abdelqadir et al. 2020 [11] | 8 | Egypt | 180 - DN | 60 | *Lactobacillus, Bifidobacterium* | Yes (Cochrane)  6/8 high | WMD | ↑ TAC  ↓ CRP, MDA  ↔ NO, GSH |
| Bohlouli et al. 2021 [12] | 4 | Iran | 240 - DN | 58 | *Lactobacillus, Bifidobacterium* | Yes (Cochrane)  1/4 high | WMD | ↑ TAC, GSH  ↓ CRP, MDA  ↔ NO |
| Wang et al. 2021 [13] | 7 | China | 240 - DN | 60 | *Lactobacillus, Bifidobacterium* | Yes (Cochrane)  7/7high | WMD | ↑ TAC, GSH  ↓ CRP, MDA  ↔ NO |
| Dai et al. 2022 [14] | 3 | China | 180 - DN | 59 | *Lactobacillus, Bifidobacterium* | Yes (Cochrane)  1/3 high | SMD: TAC  WMD: GSH, CRP, MDA, NO | ↑ TAC, GSH  ↓ CRP, MDA  ↔ NO |
| Naseri et al. 2022 [15] | 26 | Iran | 1417- Prediabetes and T2DM | 53 | *Lactobacillus, Bifidobacterium, Streptococcus* | Yes (Cochrane)  26/26 high | WMD | ↑ TAC, GSH, NO  ↓ MDA, CRP, TNF-α  ↔ IL-6 |

Abbreviations: CRP, C-reactive protein; GDM, gestational diabetes mellitus; GSH, glutathione; IL-6, interleukin-6; MDA, malondialdehyde; NO, nitric oxide; NR, not reported; SMD, standardized mean difference; T2DM, type 2 diabetes mellitus; TAC, total antioxidant capacity; TNF-α, tumor necrosis factor-alpha; WMD, weighted mean difference.

**Table S2:** Results of assess the methodological quality of meta-analysis

| Study | **Q1^1^** | **Q2** | **Q3** | **Q4** | **Q5** | **Q6** | **Q7** | **Q8** | **Q9** | **Q10** | **Q11** | **Q12** | **Q13** | **Q14** | **Q15** | **Q16** | **Quality assessment** |
| --- | --- | --- | --- | --- | --- | --- | --- | --- | --- | --- | --- | --- | --- | --- | --- | --- | --- |
| Kocsis et al et al.2020 | Yes | Yes | No | Partial Yes | Yes | Yes | No | Yes | Yes | No | Yes | Yes | Yes | Yes | Yes | Yes | Moderate |
| Yao et al.2017 | Yes | Partial Yes | No | Partial Yes | Yes | Yes | No | Yes | Yes | No | Yes | Yes | Yes | No | No | Yes | Moderate |
| Ardeshirlarijan et al.2019 | No | Yes | Yes | Partial Yes | Yes | Yes | Partial Yes | Yes | Yes | Yes | Yes | Yes | Yes | No | Yes | Yes | High |
| Hasain et al.2021 | No | Partial Yes | Yes | Partial Yes | Yes | Yes | Partial Yes | Yes | Yes | Yes | Yes | Yes | Yes | No | No | Yes | Moderate |
| Zhang et al.2019 | No | Partial Yes | Yes | Yes | Yes | Yes | Partial Yes | Yes | Yes | Yes | Yes | Yes | Yes | Yes | Yes | Yes | High |
| Tabrizi et al.2019 | Yes | Partial Yes | No | Yes | Yes | Yes | No | Yes | Yes | No | Yes | Yes | Yes | Yes | Yes | Yes | Moderate |
| Samah et al.2016 | Yes | Partial Yes | No | Partial Yes | Yes | Yes | No | Yes | Yes | Yes | Yes | Yes | Yes | Yes | Yes | Yes | Moderate |
| Chan et al.2021 | No | Partial Yes | Yes | Partial Yes | Yes | Yes | Partial Yes | Yes | Yes | No | Yes | Yes | Yes | No | No | Yes | Moderate |
| Kasińska et al.2015 | Yes | Partial Yes | No | Partial Yes | Yes | Yes | Partial Yes | Yes | Yes | No | Yes | Yes | Yes | Yes | Yes | Yes | Moderate |
| Chen et al.2020 | No | Partial Yes | Yes | Partial Yes | Yes | Yes | Yes | Yes | Yes | No | Yes | Yes | Yes | Yes | No | Yes | Moderate |
| Abdelqadir et al.2020 | No | Yes | Yes | Partial Yes | Yes | Yes | No | Partial Yes | Yes | No | No | Yes | Yes | No | No | Yes | Moderate |
| Bohlouli et al.2021 | No | Yes | Yes | Partial Yes | Yes | Yes | No | Partial Yes | Yes | No | Yes | Yes | Yes | Yes | Yes | Yes | Moderate |
| Wang et al.2021 | Yes | Yes | No | Partial Yes | Yes | Yes | No | Partial Yes | Yes | No | Yes | Yes | Yes | Yes | Yes | Yes | Moderate |
| Naseri et al.2022 | Yes | Partial Yes | Yes | Partial Yes | Yes | Yes | Yes | Yes | Yes | Yes | Yes | Yes | Yes | Yes | Yes | Yes | High |
| Dai et al.2022 | No | Yes | No | Partial Yes | No | Yes | No | Yes | Yes | No | Yes | Yes | Yes | Yes | Yes | Yes | Moderate |

^1. Did the research questions and inclusion criteria for the review include the components of PICO? 2. Did the report of the review contain an explicit statement that the review methods were established prior to the conduct of the review and did the report justify any significant deviations from the protocol? 3. Did the review authors explain their selection of the study designs for inclusion in the review? 4. Did the review authors use a comprehensive literature search strategy? 5. Did the review authors perform study selection in duplicate? 6. Did the review authors perform data extraction in duplicate? 7. Did the review authors provide a list of excluded studies and justify the exclusions? 8. Did the review authors describe the included studies in adequate detail? 9. Did the review authors use a satisfactory technique for assessing the risk of bias (RoB) in individual studies that were included in the review? 10. Did the review authors report on the sources of funding for the studies included in the review? 11. If meta-analysis was performed, did the review authors use appropriate methods for statistical combination of results? 12. If meta-analysis was performed, did the review authors assess the potential impact of RoB in individual studies on the results of the meta-analysis or other evidence synthesis? 13. Did the review authors account for RoB in individual studies when interpreting/ discussing the results of the review? 14. Did the review authors provide a satisfactory explanation for, and discussion of, any heterogeneity observed in the results of the review? 15. If they performed quantitative synthesis, did the review authors carry out an adequate investigation of publication bias (small study bias) and discuss its likely impact on the results of the review? 16. Did the review authors report any potential sources of conflict of interest, including any funding they received for conducting the review? 
Each question was answered with “Yes”, “Partial Yes” or “No”. When no meta-analysis was done, question 11, 12 and 15 were answered with “No meta-analysis conducted^

**References:**

1. Kocsis, T., et al., *Probiotics have beneficial metabolic effects in patients with type 2 diabetes mellitus: a meta-analysis of randomized clinical trials.* Sci Rep, 2020. **10**(1): p. 11787.

2. Yao, K., et al., *Effect of Probiotics on Glucose and Lipid Metabolism in Type 2 Diabetes Mellitus: A Meta-Analysis of 12 Randomized Controlled Trials.* Med Sci Monit, 2017. **23**: p. 3044-3053.

3. Tabrizi, R., et al., *The effects of probiotic and synbiotic supplementation on inflammatory markers among patients with diabetes: A systematic review and meta-analysis of randomized controlled trials.* Eur J Pharmacol, 2019. **852**: p. 254-264.

4. Hasain, Z., et al., *Diet and pre-intervention washout modifies the effects of probiotics on gestational diabetes mellitus: a comprehensive systematic review and meta-analysis of randomized controlled trials.* Nutrients, 2021. **13**(9): p. 3045.

5. Zheng, H.J., et al., *The effect of probiotic and synbiotic supplementation on biomarkers of inflammation and oxidative stress in diabetic patients: A systematic review and meta-analysis of randomized controlled trials.* Pharmacol Res, 2019. **142**: p. 303-313.

6. Ardeshirlarijani, E., et al., *Effect of probiotics supplementation on glucose and oxidative stress in type 2 diabetes mellitus: A meta-analysis of randomized trials.* DARU Journal of pharmaceutical sciences, 2019. **27**: p. 827-837.

7. Samah, S., et al., *Probiotics for the management of type 2 diabetes mellitus: A systematic review and meta-analysis.* Diabetes Res Clin Pract, 2016. **118**: p. 172-82.

8. Chan, K.Y., et al., *Dietary supplementation for gestational diabetes prevention and management: a meta-analysis of randomized controlled trials.* Arch Gynecol Obstet, 2021. **303**(6): p. 1381-1391.

9. Kasińska, M.A. and J. Drzewoski, *Effectiveness of probiotics in type 2 diabetes: a meta-analysis.* Pol Arch Med Wewn, 2015. **125**(11): p. 803-13.

10. Chen, Y., et al., *Effects of probiotics on blood glucose, biomarkers of inflammation and oxidative stress in pregnant women with gestational diabetes mellitus: A meta-analysis of randomized controlled trials.* Med Clin (Barc), 2020. **154**(6): p. 199-206.

11. AbdelQadir, Y.H., et al., *Efficacy of probiotic supplementation in patients with diabetic nephropathy: A systematic review and meta-analysis.* Clin Nutr ESPEN, 2020. **40**: p. 57-67.

12. Bohlouli, J., et al., *Effect of probiotics on oxidative stress and inflammatory status in diabetic nephropathy: A systematic review and meta-analysis of clinical trials.* Heliyon, 2021. **7**(1): p. e05925.

13. Wang, H., et al., *The effects of probiotic supplementation on renal function, inflammation, and oxidative stress in diabetic nephropathy: A systematic review and meta-analysis of randomized controlled trials.* Materials Express, 2021. **11**(7): p. 1122-1131.

14. Dai, Y., et al., *Probiotics improve renal function, glucose, lipids, inflammation and oxidative stress in diabetic kidney disease: a systematic review and meta-analysis.* Renal Failure, 2022. **44**(1): p. 862-880.

15. Naseri, K., et al., *The effects of probiotic and synbiotic supplementation on inflammation, oxidative stress, and circulating adiponectin and leptin concentration in subjects with prediabetes and type 2 diabetes mellitus: A GRADE-assessed systematic review, meta-analysis, and meta-regression of randomized clinical trials.* European Journal of Nutrition, 2023. **62**(2): p. 543-561.
